# Personality domains in early stages of psychosis: a systematic review and meta-analysis

**DOI:** 10.1007/s00406-025-02127-4

**Published:** 2025-11-12

**Authors:** Mauro Scala, Davide Gori, Pablo Roca, Chiara Fabbri, Rocío Arroyo Iturra, Sergio Felipe Calvo García, Giuseppe Fanelli, Alessandro Serretti

**Affiliations:** 1 Universidad Europea de Madrid, Faculty of Biomedical and Health Sciences, Campus de Villaviciosa, Calle Tajo s/n, 28670, Villaviciosa de Odón, Madrid, Spain; 2 School of Medicine, Complutense University of Madrid, Madrid, Spain; 3 Health Research Institute Hospital 12 de Octubre, (imas12), Madrid, Spain; 4 Department of Biomedical and Neuromotor Sciences, University of Bologna, Bologna, Italy; 5 Faculty of Health Sciences, Universidad Villanueva, Madrid, Spain; 6 Department of Experimental Psychology, Complutense University of Madrid, Madrid, Spain; 7 Donders Institute for Brain, Cognition and Behaviour, Radboud University, Nijmegen, The Netherlands; 8 Department of Human Genetics, Radboud University Medical Center, P.O. Box 9101, 6500 HB, Nijmegen, The Netherlands; 9 Department of Medicine and Surgery, Kore University of Enna, Enna, Italy; 10 Oasi Research Institute-IRCCS, Troina, Italy

**Keywords:** Early psychosis, Personality, Neuroticism, Five-factor model, Psychotic disorders

## Abstract

**Background:**

Personality traits influence symptoms, functioning, and illness trajectory in chronic psychosis. However, their role in early-stage psychosis remains poorly defined, particularly regarding potential differences from healthy controls and their association with clinical outcomes.

**Methods:**

We conducted a systematic review and meta-analysis of studies assessing personality domains in early-stage psychosis using validated dimensional instruments. Searches were performed in PubMed/MEDLINE, CINAHL, and Web of Science until March 2025. The meta-analysis included studies using the NEO Five-Factor Inventory (NEO-FFI), with patient scores compared to published normative data. Studies reporting T-scores and those reporting raw scores were analyzed separately. Associations between personality domains and clinical features were narratively synthesized.

**Results:**

Eighteen studies met the inclusion criteria; eight were included in the meta-analysis (*n* = 1109). Considering studies reporting T-scores, individuals with early-stage psychosis showed higher neuroticism (*MD* = 27.4, *95% CI* [25.0 to 29.9]) and lower extraversion (*MD* = −6.0, *95% CI* [–8.6 to −3.5]) and conscientiousness (*MD *= −5.5, *95% CI* [–7.9 to −3.2]), relative to normative data. Analyses of studies reporting raw scores showed similar effects, though not statistically significant. The same personality domains were consistently associated with symptom severity, treatment adherence, functioning, and service use.

**Conclusions:**

Early-stage psychosis may be characterized by a specific personality profile that modulates clinical presentation. Early personality assessment may guide tailored treatment strategies. Longitudinal studies are needed to clarify their prognostic relevance and potential role in the personalization of treatment.

**Supplementary Information:**

The online version contains supplementary material available at 10.1007/s00406-025-02127-4.

## Introduction

The interplay between dimensional personality traits and psychotic disorders received increasing attention over recent decades. Accumulating evidence suggests that personality domains are associated with positive and negative symptoms, psychosocial functioning, treatment adherence, and illness trajectory in schizophrenia and related psychoses [1–3].

Consequently, psychiatric nosology adopted trait-based models to capture personality variability in clinical populations. The fifth edition of the Diagnostic and Statistical Manual of Mental Disorders (DSM–5) introduced the Alternative Model for Personality Disorders (AMPD), which integrates dimensional and categorical approaches [4, 5]. The AMPD organizes maladaptive traits into five broad domains (i.e., negative affectivity, detachment, antagonism, disinhibition, and psychoticism) commonly considered maladaptive variants of the Five-Factor Model (FFM) dimensions (i.e., neuroticism, extraversion, openness to experience, agreeableness, and conscientiousness), collectively known as the “Big Five” [6, 7]. These personality domains are most commonly assessed with the NEO Personality Inventory-Revised (NEO-PI-R) or the shorter version, the NEO Five-Factor Inventory (NEO-FFI) [8]. The NEO-FFI is widely adopted due to its brevity, ease of administration, and applicability in both clinical and research settings [9, 10].

Substantial research in chronic stages of psychosis, particularly schizophrenia, documented higher neuroticism and lower extraversion, openness, agreeableness, and conscientiousness compared with healthy controls (HCs) [11, 12]. High neuroticism is consistently linked to anxiety and depressive symptoms; positive symptoms correlate with higher neuroticism and lower agreeableness, whereas negative symptoms correlate with lower extraversion [13, 14].

However, these findings refer only to chronic psychosis, raising the question of whether similar trait profiles appear in early stages of psychosis (ESP), including first-episode psychosis (FEP) or schizophrenia (FES), recent-onset psychosis (ROP) or schizophrenia (ROS), or early psychosis (EP). ESP are clinically important as they provide an opportunity for early and tailored interventions that may improve long-term outcomes [15, 16]. Personality traits may act as risk or protective factors, influencing the disorder trajectory and outcomes [11, 17, 18]. Dimensional personality assessment in ESP may also explain inter-individual variability in the duration of untreated psychosis (DUP) and adherence to pharmacological and psychological treatments [2, 19, 20].

This systematic review and meta‑analysis pursues two objectives. The primary aim is to determine and quantify whether patients with ESP (affective or non-affective) have distinctive personality domains relative to HCs. The secondary aim is to explore associations between personality domains and clinically relevant outcomes (i.e., symptoms, relapse risk, treatment adherence, therapeutic alliance, DUP, and other related measures), through a narrative synthesis of the same body of studies. Clarifying these relationships may support the development of stratified intervention strategies in ESP.

## Methods

This systematic review and meta-analysis followed the Preferred Reporting Items for Systematic Reviews and Meta-Analyses (PRISMA) guidelines [21]. The protocol was registered on the International Prospective Register of Systematic Reviews (PROSPERO) (CRD42024586667). Minor deviations from the original protocol are outlined in the Supplementary Materials, Appendix I.

### Search strategy

We systematically searched the PubMed/MEDLINE, CINAHL, and Web of Science databases from inception until March 2025, using Medical Subject Headings (MeSH) and free-text terms related to personality traits and dimensions, as well as to early psychosis. Search queries are provided in Supplementary Table S1. References of all included studies were hand-searched to identify additional eligible articles.

### Eligibility criteria

Studies were eligible if they: (1) were peer-reviewed English-language articles; (2) included adult patients (18–65 years) who met at least one of the following: (a) FEP (affective or non-affective) within the last three years, a time-window commonly adopted in early-intervention services and multicenter research cohorts [22, 23]; (b) FES, ROP, ROS, or EP within three years of onset; (c) first contact with clinical services or first psychiatric admission for psychosis; or (d) explicit author designation as FEP, FES, ROP, ROS or EP regardless of a stated temporal threshold, to ensure ecological validity and reflect real-world variability in operational definitions; and 3) assessed personality, character, or temperament using validated dimensional measures, such as the NEO Personality Inventory-Revised (NEO-PI-R) or the shorter NEO Five-Factor Inventory (NEO-FFI) [8], the Tridimensional Personality Questionnaire (TPQ) [24], the Eysenck Personality Questionnaire (EPQ) [25], the Zuckerman-Kuhlman Personality Questionnaire (ZKPQ) [26], the Temperament and Character Inventory (TCI) [27], the Schizotypal Personality Questionnaire (SPQ) [28] or the Big Five Inventory—10 Item Scale (BFI-10) [29, 30]. Supplementary Table S2 details these instruments.

Exclusion criteria were: (1) samples at clinical-high risk for psychosis (CHR-P) [31], or with a chronic psychotic disorder [32]; and (2) non-original studies (i.e., books, dissertations, editorials, letters to the editors, corrigendum, literature reviews, meta-analyses, case reports, clinical vignettes, proceedings, theoretical articles, qualitative studies, grey literature, etc.).

### Study selection and data extraction

All identified records were imported into EndNote, where duplicates were removed. Two authors (M.S. and S.F.C.G.) independently screened titles and abstracts. A third author (A.S.) resolved disagreements. Two reviewers (M.S. and R.A.I.) independently extracted data, including first author, publication year, country, study design, sample characteristics, sex, age, education, DUP, diagnosis, and psychometric tool used (mean ± SD for each personality domain). We also recorded measures of psychotic symptoms, type of psychopharmacological treatment, and primary outcomes related to personality profiles or correlations between personality traits and clinical features.

### Methodological quality appraisal

Two researchers (D.G. and M.S.) independently assessed the risk of bias. A third author (A.S.) resolved any disagreements. Randomized controlled trials (RCTs) were evaluated using Version 2 of the Cochrane Risk-of-Bias Tool, while observational studies were assessed with the STrengthening the Reporting of Observational Studies in Epidemiology (STROBE) Statement Checklists [33]. Each checklist item that was adequately reported counted one point (maximum = 34). A total score of ≤ 17 points (≤ 50% of items) was interpreted as low methodological quality; scores >17 points were considered intermediate/high quality.

### Statistical analysis

Only studies using the NEO-FFI [8] were meta-analyzed, as it was the most commonly used instrument. As two Likert scales (1–5 and 0–4) were used, we harmonized raw domain scores by subtracting 12 points from the 1–5 scale (range 12–60) to align with the 0–4 scale (range 0–48), as recommended by standard psychometric practice [34]. As an additional sensitivity analysis, we repeated the meta-analyses separately for studies using the 0–4 and 1–5 Likert response formats, to assess the potential impact of scoring differences on pooled estimates. Studies reporting T-scores were analyzed separately, as conversion between raw and standardized scores was not feasible due to the lack of normative references and individual-level data. Additionally, standardized mean differences (SMDs) could not be calculated because most studies lacked HC groups, precluding valid between-group comparisons. Only one study included its own HC group, therefore normative data from the NEO-FFI manual (*N* = 4000) were used to estimate HC group metrics [8]. These normative raw scores were standardized to match each study’s scoring scheme. We assessed heterogeneity with Cochran’s *Q* and the *I²* statistic. Given the expected heterogeneity across studies, random-effects models were applied. For analyses including fewer than three studies (k ≤ 2), we applied fixed-effect models, in line with methodological recommendations for meta-analyses with very low study counts [35, 36]. Funnel plots for each analysis inspected publication bias; statistical tests were not performed because fewer than ten studies were available per analysis. Sensitivity analyses included high-quality studies only. Meta-regression was not feasible because only one study included an HC group, and for the others, we used population reference data. A narrative synthesis was provided for studies using other personality scales and those examining associations between NEO-FFI and clinical outcomes.

## Results

The initial search yielded 3026 records: 1057 from PubMed/MEDLINE, 733 from Web of Science, and 1236 from CINAHL. After removing duplications, 2303 records were screened, of which 2214 articles were excluded at the title/abstract level, and 71 studies did not meet the inclusion/exclusion criteria after full-text review. Therefore, 18 studies were included in the present systematic review [2, 19, 37–52]. Among these, eight studies reporting means and standard deviations (SDs) for NEO-FFI domains were eligible for inclusion in the meta-analysis: six used raw scores [19, 37, 40, 41, 46, 51], and two used T-scores [42, 47]. A total of 1109 patients with ESP were included in the meta-analysis.

**Fig. 1:**
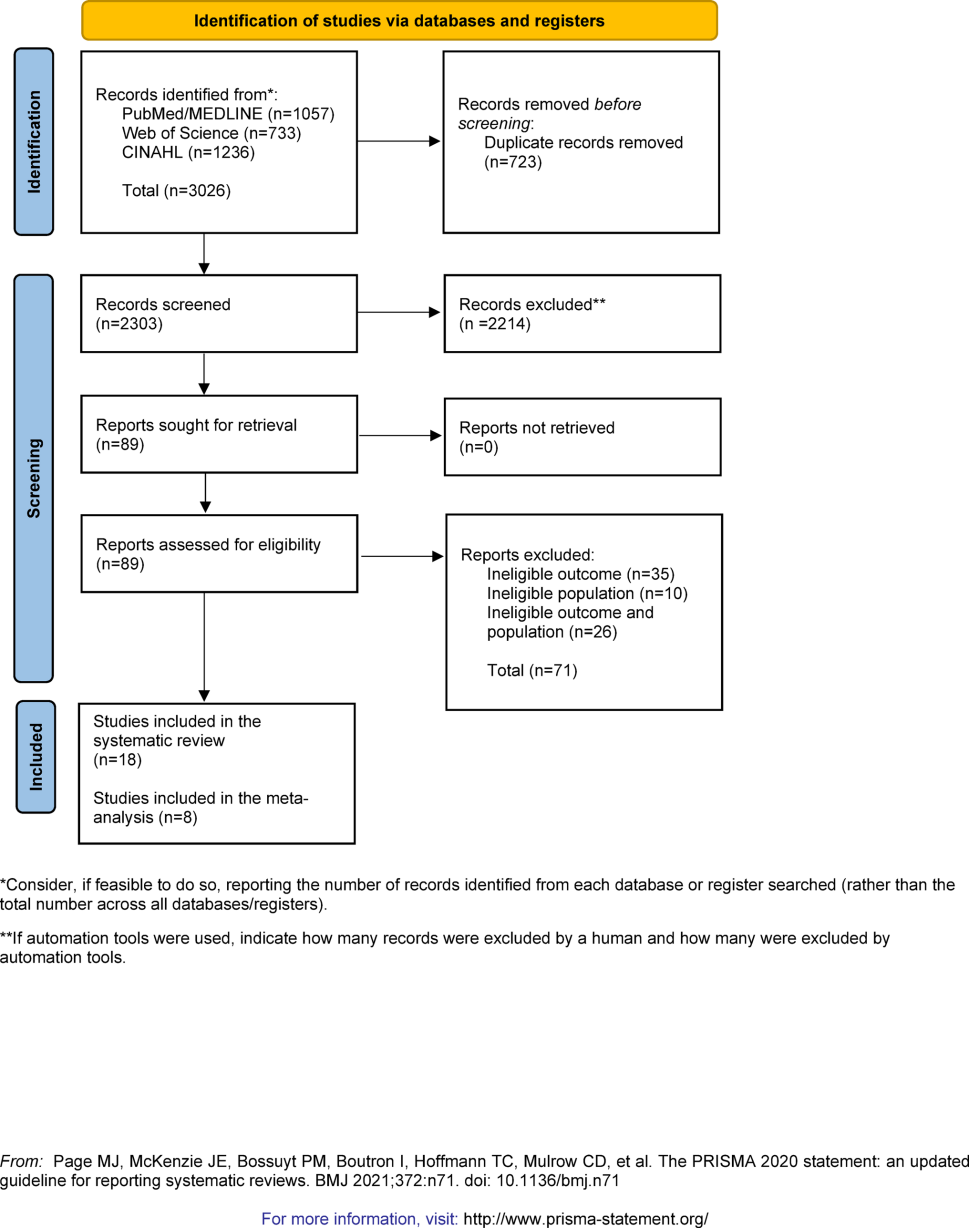
PRISMA PRISMA flow diagram for systematic review on literature search and inclusion process

The PRISMA-compliant study selection process is illustrated in Fig. 1. Studies excluded at the full-text review level, including the reasons for exclusion, are listed in Supplementary Table S3. Studies using the NEO-FFI but excluded from the meta-analysis due to missing descriptive statistics (i.e., means and SDs) or overlapping samples are listed in Supplementary Table S4. Details on the included studies are provided in Table 1, while Table 2 outlines the operational definitions of FEP, FES, ROP, ROS, and EP used by the original authors. Results are presented in the following paragraphs according to the two review objectives, with quantitative synthesis addressing personality differences between ESP and controls, and narrative synthesis examining associations with clinical outcomes.

**Table 1:** Summary of included studies published in peer-reviewed journals until March 2025

First author, year	Location/ ethnicity or race (%)	Study type	Sample size (n)	Sex (F/M%)	Age (mean ± SD)	Education (yrs)	DUP mths (mean ± SD)	Diagnosis (n or %)		Personality measures (mean ± SD) by psychometric tool and domain	Psychopathological measure (mean ± SD)/ pharmacotherapy	Personality domain profiles and their associations with clinical outcomes	
Strakowski S et al., (1992)	USA	Prospective cohort study	FEP 61*	54.1/45.9	33.6 ± 13.2	NA	NA	BD 38 (manic 27, mixed 11) MDD 9 P-NOS 5 DD 4 SFD 3 SAD 1 BPD 1		TPQ *Novelty seeking* Manic BD 15.6 ± 4.3 Mixed BD 18.9 ± 6.6 MDD 13.0 ± 5.9 *Harm avoidance* Manic BD 11.3 ± 8.6 Mixed BD 17.7 ± 7.2 MDD 17.4 ± 6.6 *Reward dependence* Manic BD 20.8 ± 3.4 Mixed BD 15.6 ± 4.3 MDD 19.8 ± 2.1	BPRS Mania 6.6 ± 3.2 Depression 4.3 ± 1.6 Total 41.3 ± 8.7	PERSONALITY AND CLINICAL FEATURES *harm avoidance* Positively correlates with depressive symptoms (*r* = 0.42, *p* < 0.001) Negatively correlates with manic symptoms (*r*= -0.34, *p* < 0.01) *	
	White 83.6%										NA		
Wolthaus J et al., (2002)	The Netherlands	LS	ROS 138 CG 103	23.2/76.8	23.2 ± 5.3	70.3%≥12 29.7%<9 **	NA	SCZ 104 SFD 20 SAD 14		NEO-FFI N 24.2 ± 8.4 E 25.3 ± 7.3 O 25.8 ± 5.2 A 29.5 ± 4.9 C 27.7 ± 6.0 SPQ Positive schizotypy 12.6 ± 8.5 Negative schizotypy 15.4 ± 9.6 Disorganization 6.1 ± 5.2	PANSS components Positive 18.0 ± 7.3 Negative 16.6 ± 6.5 Depression 9.7 ± 4.0 Agitation-excitement 6.4 ± 2.8 Disorganization 13.7 ± 5.0	PERSONALITY AND CLINICAL FEATURES Personality traits (NEO-FFI) not predictive of caregiver burden (IEQ total and subscales: supervision, tension, worrying, and urging) Disorganization (SPQ) predicted caregiver worrying (IEQ i.e., concern about patients’s safety), beyond PANSS agitation (ΔR²=0.06, *p* = 0.045).	
	NA										Antipsychotics for at least 6 weeks without switching		
Gleeson et al., (2005)	Australia	LS	FEP 60 American norms 389	NA	22.5 ± 3.0	NA	NA	SCZ 23 SFD 8 SAD 3 DD 1 BD 11 MDD 11 P-NOS 2 BPD 1		NEO-PI-R N 107.6 ± 22.6 E 100.5 ± 21.2 O 113.0 ± 15.9 A 118.6 ± 14.9 C 99.8 ± 20.2 *Personality assessment was conducted on 40 FEPs*	NA	PERSONALITY PROFILE FEP vs. American norms ↑ N (d = 0.51, *p* = 0.002) and A (d = 0.31, *p* = 0.029) ↓ E (*p* = 0.002, *p* < 0.001) and C (d = 0.70, *p* < 0.001)	
	NA										No more than 6 mths of previous treatment with antipsychotics	PERSONALITY AND CLINICAL FEATURES ↓ A domain and facets (trust [d = 0.73, *p* = 0.026], altruism [d = 0.87, *p* = 0.009]) predict relapse	
Beauchamp et al., (2006)	Canada	CS	FEP 79 HC 66	27.0/73.0	24.0 ± 4.5	13 ± 2.8	NA	SCZ 38.3% SAD 18.5% BD 4.9% MDD 6.2% SFD 2.5% BPD 2.5% P-NOS 27.1%		NEO-PI-R N 35.1 ± 4.5 E 38.0 ± 5.5 O 37.7 ± 4.4 A 38.1 ± 4.0 C 39.6 ± 4.3	BPRS Total 41.5 ± 12.0	PERSONALITY PROFILE FEP vs. HC ↑ N (*p* < 0.001, ES = 0.76), O (*p* < 0.01, ES = 0.70), and A (*p* < 0.001, ES = 0.65) ↓ E (*p* < 0.001, ES = 1.27) and C (*p* < 0.01, ES = 0.59).	
	White 58% Asian 13% First Nations 4% Latin American 3% African/Caribbean 1% Other 22%										NA	PERSONALITY AND CLINICAL FEATURES FEPs with the lowest E, O, and C show ↑ levels of suspiciousness (*p* < 0.05) and unusual thought content (*p* < 0.05)	
Couture et al., (2007)	Canada	CS	FEP 96 Beauchamp et al. (2006) HC 66 *for personality trait analyses* Paquette et al. (2001) HC 353 *for attachment analyses*	33.7/66.3	23.7 ± 4.7	12.4 ± 2.1	NA	SSD 96		NEO-FFI NA	BPRS Total 40.9 ± 13.2	PERSONALITY PROFILE FEP vs. HC ↑ O (F[1,152] = 8.28, *p* < 0.01) and A (F[1,152] = 12.52, *p* < 0.01) Trend toward ↑ N (F[1,152] = 5.89, *p* = 0.016) ↓ E (F[1,152] = 34.3, *p* < 0.001)	
	White 59.6% Asian 18.1% Other 16.0%										NA	PERSONALITY AND CLINICAL FEATURES ↑ A associated with ↓ inappropriate behaviors (β= − 0.289, *p* < 0.05); ↑ N trend-level association with ↓ quality of life (β=–0.201, *p* = 0.085)	
Lecomte et al., (2008)	Canada	CS	EP 118	39.0/61.0	25.0 ± 5.9	12 ± 2.3	NA	SCZ 66 SAD 27 BD 13 P-NOS 12		NEO-FFI N 34.6 ± 4.8 E 40.5 ± 6.5 O 37.6 ± 4.9 A 38.1 ± 5.5 C 40.7 ± 5.7	BPRS Total 43.9 ± 14.3 Positive 2.0 ± 0.9 Negative 1.6 ± 0.7	PPERSONALITY AND CLINICAL FEATURES ↑ A predicts poor medication adherence (OR. 1.1, *p* < 0.05) ↑ N (β= -0.19, *p* < 0.01) and ↓ A (β = 0.25, p = *p* < 0.01) predict good service engagement	
	Caucasian 60% Asian 16% First Nations 5%										Atypical antipsychotics 100%. Mood stabilizers in augmentation with antipsychotics		
Beauchamp M et al., (2011)	Canada	RCT	FEP 129 Waiting list 66	27.0/73.0	24.0 ± 5.2	12 ± 2.0	NA	SSD 66 BD or MDD 25 BPD or P-NOS 38		NEO-FFI N 35.8 ± 4.7 E 37.8 ± 5.1 O 36.9 ± 4.2 A 38.6 ± 5.3 C 39.9 ± 4.1	BPRS Positive 2.1 Negative 1.9 Total 42.3	PERSONALITY AND CLINICAL FEATURES No correlation between personality dimensions and psychotic symptoms All traits significantly correlate with improvements in active coping N (*r* = 0.257, *p* < 0.05), E (*r* = 0.411, *p* < 0.01), O (*r* = 0.324, *p* < 0.01), A (*r* = 0.275, *p* < 0.05), C (*r* = 0.368, *p* < 0.01). N positively correlates with passive coping (*r* = 0.245, *p* < 0.05)	
	Caucasian 61% Asian 9% Aboriginal 3%										The large majority of the participants were treated with novel antipsychotics		
Ruiz-Veguilla et al., (2012)	Spain	Retrospective study	FEP 97	44/56	30.7 ± 9.1	8.5 ± 3.8	6.2 ± 13.7	non-affective psychosis 73		EPQ N 14.6 ± 5.6 E 11.1 ± 4.0	NA	PERSONALITY AND CLINICAL FEATURES In FEPs with good diffuse social support, ↑ N is associated with longer DUP In FEPs with poor social support, ↓ N is associated with longer DUP	
	NA										Drug-naïve		
Beauchamp M et al., (2013)	Canada	RCT	FEP 129	23.6/76.5	23.7 ± 5.0	12.6	NA	SSD 73 MDD 25 P-NOS 30		NEO-FFI N 35.8 ± 4.7 E 37.8 ± 5.1 O 36.9 ± 4.2 A 38.6 ± 5.3 C 41.3 ± 3.2	BPRS NA	PERSONALITY AND CLINICAL FEATURES Improvement in positive symptoms negatively correlates with A (*r*= -0.315, *p* < 0.05) and O (*r*= -0.296, *p* < 0.05) Improvement in negative symptoms negatively correlates with C (*r*= -0.677, *p* < 0.05) *Significant bivariate correlations found*,* but no predictive value in regression analysis* Improvement in active coping positively correlates with C (*r* = 0.379, *p* < 0.05), E (*r* = 0.637, *p* < 0.01), O (*r* = 0.452–0.471, *p* < 0.05), A (*r* = 0.486, *p* < 0.05) Improvement in passive coping positively correlates with O (*r* = 0.441, *p* < 0.05) *correlation analyses were performed on 78 FEPs*	
	White 66.7% Asian 9.3% First Nations 3.1% Other 19.4%										NA		
Johansen R et al., (2013)	Norway	CS	ROS 42	33.3/66.7	27.5 ± 5.6	11.8 ± 1.9	NA	SCZ 36 SAD 3 DD 3		NEO-FFI N 60.4 ± 10.1 E 40.0 ± 9.3 O 42.4 ± 6.8 A 50.0 ± 10.4 C 42.2 ± 8.3	PANSS 66.9 ± 15.6 Positive 17.0 ± 5.6 Negative 15.4 ± 6.1 Depression 13.4 ± 4.5 Agitation-excitement 5.9 ± 2.2 Disorganization 13.1 ± 4.3	PERSONALITY AND CLINICAL FEATURES *Therapeutic alliance – patient ratings* A positively correlates with therapeutic alliance (WAI-S patient total: ρ = 0.317, *p* < 0.05) N negatively correlates with therapeutic alliance (WAI-S patient total: ρ=−0.325, *p* < 0.05) *Therapeutic alliance – therapist ratings* A and PANSS insight item (G12) predict 23% of the variance in therapist-rated alliance	
	NA										Antipsychotics 78.6% Mood stabilizers 9.5% Antidepressants only 2.4% No medication 19.0%		
Song YY et al., (2013)	South Korea	LS	FES 33 UHR 50 HC 120	57.6/42.4	21.4 ± 3.6	13.3 ± 2.3	9.1 ± 9.7	SCZ 33		TCI Temperament Novelty seeking 53.8 ± 9.2 Harm avoidance 56.6 ± 12.8 Reward dependence 43.3 ± 11.7 Persistence 44.2 ± 12.0 Character Self-directedness 42.5 ± 11.6 Cooperativeness 44.1 ± 14.0 Self-transcendence 49.8 ± 9.8	SAPS 6.9 ± 2.6 SANS 7.9 ± 4.4	PERSONALITY PROFILE FES vs. HC ↑ *harm avoidance* (F_2,200_= 31.7, *p* < 0.001) ↓ *reward dependence* (F_2,200_= 27.8, *p* < 0.001), ↓ *persistence* (F_2,200_=13.3, *p* < 0.001), ↓ *self-directedness* (F_2,200_=24.9, *p* < 0.001), ↓ *cooperativeness* (F_2,200_=7.3, *p* = 0.001) No differences in *self-transcendence* (F_2,200_=0.7, *p* = 0.483) PERSONALITY AND CLINICAL FEATURES No correlation between personality dimensions and psychotic symptoms ↑ social functioning (GF: Social) correlates with: ↓ *harm avoidance* (*r*=–0.56) ↑ *self-directedness* (*r* = 0.59) ↑ self-esteem (Rosenberg’s Self-Esteem Scale) correlates with: ↓ *harm avoidance* (*r*=–0.71) ↑ *self-directedness* (*r* = 0.71) ↑ general self-efficacy (The self-efficacy Scale) correlates with: ↓ *harm avoidance* (*r*=–0.82) ↑ *persistence* (*r* = 0.57) ↑ *self-directedness* (*r* = 0.70) ↑ social self-efficacy (The self-efficacy Scale) correlates with: ↓ *harm avoidance* (*r*=–0.82) ↑ *persistence* (*r* = 0.58) ↑ *self-directedness* (*r* = 0.73)	
	NA										Antipsychotics 97.0% CLPZ equivalent dose 461.1 ± 307.2		
Bozidis et al., (2014)	Greece	CS	FES 50 HC 50	32.0/68.0	29.8 ± 8.2	24% = 5 30% = 9 46% ≥12**	23.9 ± 38.5	SCZ 39 DD 5 APPD 6		ZKPQ Impulsive Sensation Seeking 8.93 ± 3.60 N 10.3 ± 3.9 Aggression–Hostility 7.13 ± 2.22 Activity 9.10 ± 2.18 Sociability 6.80 ± 2.16	PANSS Total 98.7 ± 20.5	PERSONALITY PROFILE FES vs. HC ↑ N (*p* < 0.001) ↑ aggression–hostility (*p* = 0.023) ↓ sociability (*p* < 0.001)	
	Greek 100%										Drug-naïve		
Gurrera et al., (2014)	USA	CS	FES 30 HC 62	70.0/30.0	25.2 ± 6.9	14.0 ± 2.1	NA	SCZ 30		NEO-FFI N 58.9 ± 10.3 E 44.6 ± 15.0 O 56.0 ± 10.4 A 46.9 ± 13.0 C 39.8 ± 15.5	PANSS Positive 20.8 ± 4.0 Negative 17.6 ± 7.2 General 35.2 ± 7.9 Depression 9.5 ± 4.3	PERSONALITY PROFILE FES vs. HC ↑ N (F = 72.701, *p* < 0.001) ↓ E (F = 21.995, *p* < 0.001) ↓ A (F = 8.821, *p* = 0.004) ↓ C (F = 19.076, *p* < 0.001) ↓O (F = 4.600, *p* = 0.035) PERSONALITY AND CLINICAL FEATURES ↑ Negative symptoms correlate with: ↑ N (*r* = 0.471, *p* = 0.009) ↓ C (*r*=–0.464, *p* = 0.010) No correlations betwenn positive and general symptoms with to personality traits. ↑ Attention/planning correlates with: ↑ E (*r* = 0.491, *p* = 0.006) ↑ C (*r* = 0.619, *p* = 0.000) ↑ Memory correlates with: ↑ O (*r* = 0.540, *p* = 0.002)	
	White 76.7% Black 20.0% Pacific Islander 3.3%										Drug-naïve		
Compton et al., (2015)	USA	CS	FEP 104	28.9/71.2	24.5 ± 5.2	11.9 ± 2.0	12.3	SCZ 57 P-NOS 16 SFD 12 SAD 15 BPD 2 DD 2		NEO-FFI N 21.6 ± 8.6 E 29.5 ± 6.7 O 27.0 ± 4.7 A 28.3 ± 6.7 C 33.4 ± 7.6	PANSS Positive 22.7 ± 5.0 Negative 21.2 ± 6.1	PERSONALITY AND CLINICAL FEATURES *DUP* ↑ N (*r* = 0.38, *p* < 0.001) ↓ E (*r*=–0.32, *p* = 0.001) ↓ A (*r*=–0.29, *p* = 0.003) ↓ C (*r*=–0.22, *p* = 0.023) In multiple regression, N (β = 0.29, *p* = 0.010), E (β=–0.26, *p* = 0.035), and A (β=–0.20, *p* = 0.049) remained significant predictors of DUP. Personality domains explained 21% of DUP variance (R²=0.21, *p* < 0.001). *FUNCTIONING (GAF + SOFAS)* ↑ C (*r* = 0.37, *p* < 0.001) ↑ E (*r* = 0.31, *p* = 0.002) ↑ A (*r* = 0.25, *p* = 0.009) ↓ N (*r*=–0.28, *p* = 0.004) No significant predictors in multiple regression. Personality domains explained 16% of variance (R²=0.16, *p* = 0.003). *Positive symptoms (PANSS)* ↓ A (*r*=–0.38, *p* < 0.001; β=–0.45, *p* < 0.001) Personality domains explained 18% of positive symptom variance (R²=0.18, *p* = 0.002). *Negative symptoms (PANSS)* ↓ E (*r*=–0.28, *p* = 0.005; β=–0.25, *p* = 0.05) ↓ C (*r*=–0.23, *p* = 0.02) N was marginal (β=–0.21, *p* = 0.07) Model explained 12% of variance (R²=0.12, *p* = 0.032) A negatively correlates with risperidone dosage (*r* = − 0.25, *p* = 0.04)	
	Black/African American 85.6% White 6.7% Other 7.7%										Risperidone 68.3% daily dosage 3.9 ± 1.7 mg		
Scholte-Stalenhoef et al., (2016)	The Netherlands	Cohort study	ROP 208	29.3/70.7	28.1 ± 9.2	NA	NA	SCZ 55.3% SFD 10.1% SAD 3.8% DD 4.8% BPD 12.5% P-NOS 12.5%		NEO-FFI N 37.7 ± 8.3 E 35.2 ± 7.0 O 36.6 ± 6.0 A 41.5 ± 5.6 C 39.3 ± 7.0	PANSS Positive 12.0 ± 4.4 Negative 13.7 ± 5.0 General 28.7 ± 7.0 MADRS 12.5 ± 8.1	PERSONALITY AND CLINICAL FEATURES O positively associated with ambulatory care use (β = 0.03, SE = 0.011, *p* = 0.0057) N positively correlates with depressive symptoms (β = 5.73, *p* < 0.001) positive symptoms ↓ A (β=–4.90, SE = 1.007, *p* < 0.001) negative symptoms ↓ E (β=–4.27, SE = 1.100, *p* < 0.001) N ↓ active coping (β=–0.07, *p* = 0.0145), ↑ passive reacting (β = 0.34, *p* < 0.001) No direct effect of personality on hospitalization or day care	
	NA										NA		
Crabtree et al., (2019)	Australia	CS	EP 21 CC 55 NCC 24	57.1/42.9	25.4 ± 4.2	NA	NA	NA		NEO-FFI N 30.4 ± 8.6 E 29.3 ± 7.9 O 33.9 ± 7.2 A 28.1 ± 5.3 C 26.8 ± 7.5	P/SQ Total Paranoia/ Suspicious 24.9 ± 9.2 Interpersonal Suspicious 6.6 ± 3.4 Negative Mood 4.5 ± 1.7 Anger/ Impulsiveness 4.3 ± 3.2 Mistrust/ Wariness 3.6 ± 2.0 Hardship 3.5 ± 2.2	PERSONALITY PROFILE N EP > CC > NCC (F_2,99_ = 15.90, *p* < 0.001) O EP & CC > NCC (F_2,99_ = 17.90, *p* < 0.001) C NCC > EP (F_2,99_ = 3.45, *p* < 0.05)	
	NA										NA		
Jo A et al., (2021)	South Korea	CS	FES 169	59.2/40.8	27.4 ± 8.6	NA	12.0 ± 21.9	SCZ 115 SFD 36 other PD + SSD 14 SAD 4		BFI-10 N 3.1 ± 0.8 E 2.9 ± 0.8 O 3.5 ± 0.9 A 3.4 ± 0.8 C 3.1 ± 0.7	PANSS Positive 15.2 ± 4.6 Negative 17.1 ± 4.4 General 34.6 ± 7.3 Total 66.9 ± 14.0	PERSONALITY AND CLINICAL FEATURES C positively correlates with longer DUP (OR 2.015, *p* = 0.007)	
	NA										Pharmacotherapy 100%		
Djordjevic et al., (2022)	The Netherlands	Cohort study	ROP 527	27.1/72.9	27.6 ± 8.6	20.5%=5 42.5%=9 37.0%≥12**	82 < 1mth 109 1–3 mths 336 >3 mths	SCZ 261 SFD 59 SAD 26 DD 24 SIP 26 P-NOS 131		NEO-FFI N 37.8 ± 8.3 E 35.6 ± 7.2 O 37.1 ± 6.0 A 41.6 ± 5.6 C 39.4 ± 6.5	PANSS Positive 12.9 ± 4.8 Negative 14.3 ± 5.8 General 29.8 ± 7.9 MADRS 13.2 ± 8.6	PERSONALITY AND CLINICAL FEATURES A (B= -0.005, *p* < 0.009) and N (B= -0005, *p* < 0.005) negatively correlate with symptom severity. The association between N and PANSS is confounded by MADRS	
	Dutch 86.3%										Antipsychotics 85.6%		

Data refer exclusively to individuals with recent-onset psychosis (i.e., EP, FEP, FES, ROP, or ROS). Only outcomes directly related to dimensional personality traits were extracted and reported

*Data refer to a diagnostically mixed cohort of patients with either psychotic or manic symptoms at first psychiatric hospitalization. Results could not be disaggregated for patients with psychotic symptoms only

**The educational years for these studies are inferred from reported categories (i.e., low, medium, high, elementary school, secondary school, high school, university) rather than specific years and may have a margin of error

A = Agreeableness; APPD = acute polymorphic psychotic disorder; BD = bipolar disorder; BFI-10 = Big Five Inventory − 10 Item Scale; BPD = brief psychotic disorder; BPRS = brief psychiatric rating scale; C = Conscientiousness; CC = creative control; CS = cross sectional; CG = caregiver; CLPZ = Chlorpromazine; DD = delusional disorder; DUP = duration of untreated psychosis; E = extraversion; EF = effect size; EP = early psychosis; EPQ = Eysenck Personality Questionnaire; FEP = first episode of psycosis; FES = first episode of schizophrenia; GAF = Global Assessment of Functioning; GF = Global Functioning; HC = healthy control; IEQ = Involvement Evaluation Questionnaire; LG = longitudinal; MADRS = Montgomery-Asberg depression rating scale; MDD = major depressive disorder; mth = month; N = neuroticism; NA = not available; NCC = non-creative control; NEO-FFI = NEO five-factor inventory; O = openness; PANSS = positive and negative syndrome scale; PD = psychotic disorder; P-NOS = psycosis not-otherwise-specified; P/SQ = Paranoia/Suspiciousness Questionnaire; RCT = randomized controlled trial; ROP = recent-onset psychosis; ROS = recent-onset schizophrenia; SAD = schizoaffective disorder; SANS = Scale for the Assessment of Negative Symptoms; SAPS = Scale for the Assessment of Positive Symptoms; SCZ = schizophrenia; SFD = schizophreniform sisorder; SIP = substance-induced psychosis; SOFAS = Social and Occupational Functioning Assessment Scale; SPQ = Schizotypal Personality Questionnaire; SSD = schizophrenia spectrum disorders; TCI = Temperament and Character Inventory; TPQ = tridimensional personality questionnaire; UHR = ultra-high risk for psychosis; yrs = years; WAI-S = Working Alliance Inventory – short form; ZKPQ = five-factor Zuckerman–Kuhlman personality questionnaire

**Table 2:** Operational definitions used by the authors of the included studies to define First-Episode psychosis (FEP), first Epsiode of schizophrenia (FES), Recent-Onset psychosis (ROP), Recent-Onset schizophrenia (ROS), or early psychosis (EP)

First author, year	Included patients
Strakowski et al., (1992)	Patients with first psychiatric hospitalization with psychotic or manic symptoms. *It was not possible to extract data specific to patients with psychotic symptoms*,* as the sample included individuals hospitalized for either psychotic or manic symptoms. Hence*,* the data and the outcome refer to a diagnostically mixed cohort and may also include patients with manic episodes without psychotic features.*
Wolthaus et al., (2002)	Patients with recent-onset schizophrenia (ROS), defined as patients with a first or second psychotic episode of schizophrenia.
Gleeson et al., (2005)	Patients with a first episode of a psychotic disorder (FEP), defined as more than 1 week of active psychotic symptoms with not more than 6 months of previous treatment with antipsychotic medication.
Beauchamp et al., (2006)	Patients with a first episode of psychosis (FEP) within the past 2 years.
Couture et al., (2007)	Patients with a diagnosis of a schizophrenia spectrum disorder (SSD), and with their first episode of psychosis (FEP) within the past 2 years.
Lecompte et al., (2008)	Patients with an early psychosis (EP), defined as having consulted a medical professional for psychotic symptoms for the first time within the past 2 years.
Beauchamp et al., (2011)	Patients with a first episode of psychosis (FEP) within the past 2 years.
Ruiz-Veguilla et al., (2012)	Patients with a first episode of psychosis (FEP).
Beauchamp et al., (2013)	Patients with a first episode of psychosis (FEP) within the past two years.
Johansen et al., (2013)	Patients with recent-onset schizophrenia (ROS), defined as a diagnosis of a schizophrenia spectrum disorder (SSD) within the past two years, and ≤ 1 year after initiating contact with their current therapist.
Song et al., (2013)	Patients with first-episode schizophrenia (FES), defined as having a duration of illness ≤ 3 years from psychosis onset and no more than twelve months of antipsychotic treatment exposure.
Bozidis et al., (2014)	Patients with a first episode of schizophrenia spectrum disorders (FES), presenting for the first time to an early intervention service.
Gurrera et al., (2014)	Patients with a first episode of schizophrenia (FES) occurred within 1 year of the first hospitalization for psychotic symptoms.
Compton et al., (2015)	Patients hospitalized for a first-episode, non-affective psychotic disorder (FEP).
Scholte-Stalenhoef et al., (2016)	Patients with a first episode of non affective-psychosis (FEP).
Crabtree et al., (2019)	Patients diagnosed with an early psychosis (EP).
Jo et al., (2020)	Patients with a first episode of schizophrenia spectrum disorders (FES), who had been treated for psychotic symptoms for ≤ 2 years.
Djordjevic et al., (2022)	Patients with recent-onset psychosis (ROP), defined as experiencing psychotic symptoms for < 2 years.

### Personality traits and dimensions

Six studies reporting raw scores on the NEO-FFI showed marked heterogeneity (*I²*=100%, all Cochran’s *Q* tests *p* < 0.001). No statistically significant differences emerged between ESP and HC across the five personality domains. Pooled estimates trended toward higher neuroticism, lower extraversion, and lower conscientiousness in ESP. Sensitivity analyses separating studies by Likert response format (0–4 vs. 1–5) showed no substantial change in the overall conclusions, although minor differences emerged in the two-study subgroup using the 0–4 scale. Given the small number of studies, these differences should be interpreted with caution, as the observed significance may not represent a reliable or stable effect. Detailed findings are provided in the Supplementary Figure S1.

**Fig. 2:**
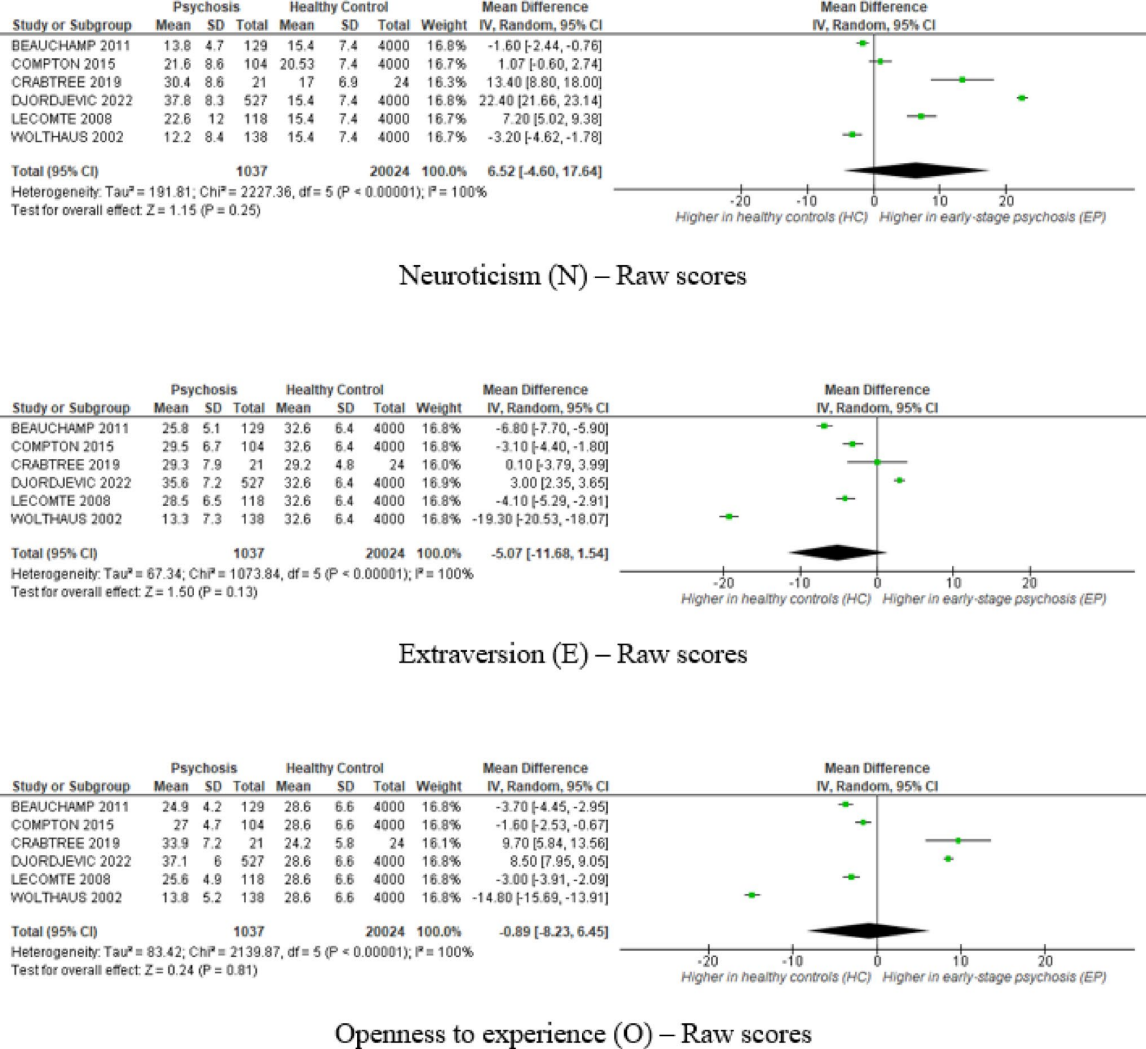

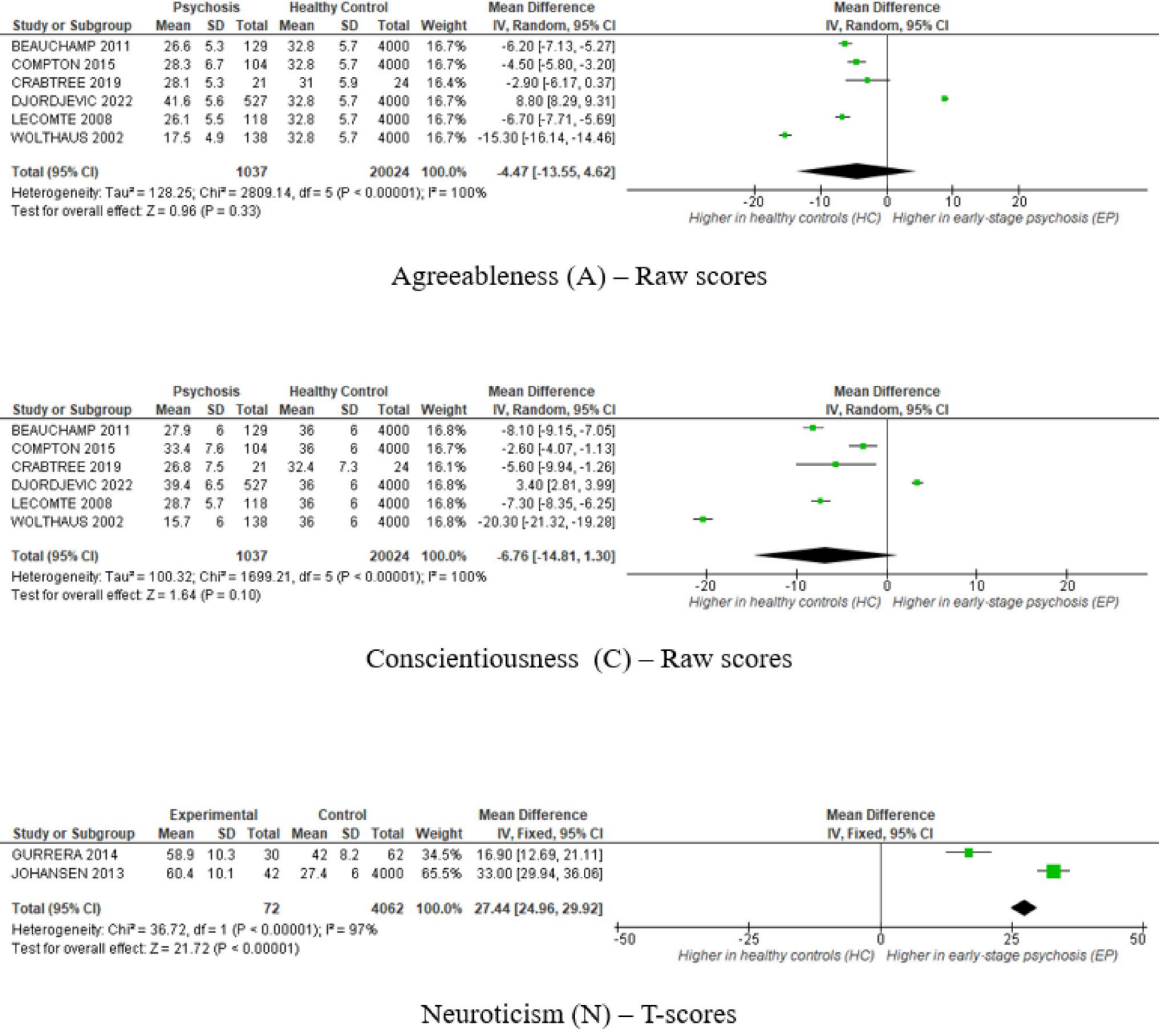

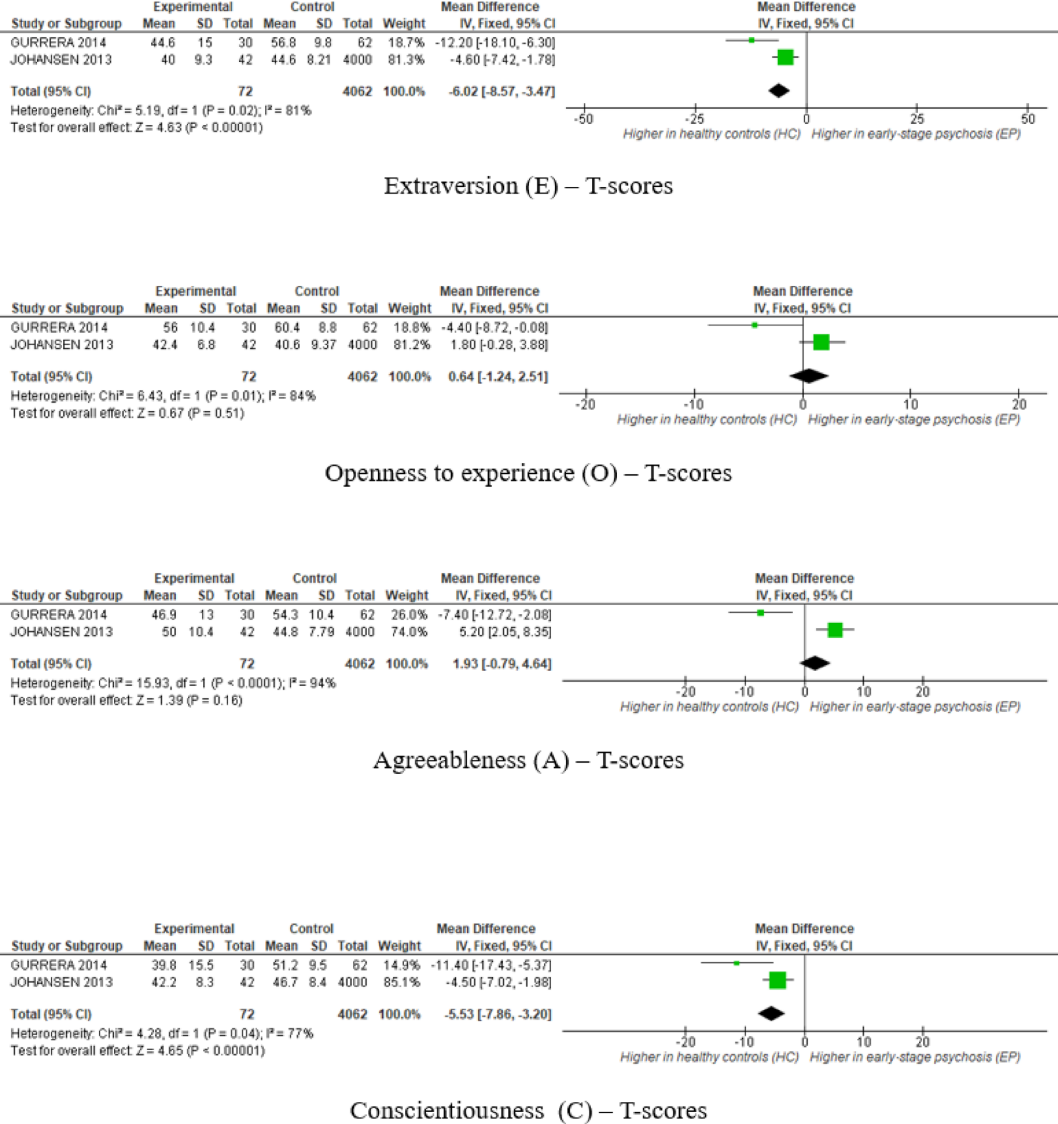
Meta-analytic estimates of personality domain differences between early-stage psychosis (ESP) and healthy controls (HC) across the five NEO-FFI domains: Neuroticism (N), Extraversion (E), Openness to Experience (O), Agreeableness (A), and Conscientiousness (C). Upper panels present analyses based on raw scores (random-effects models); lower panels show standardized T-score analyses (fixed-effects models). Positive *MD* values indicate higher scores in ESP relative to HC norms

In the T-score subgroup (k = 2), fixed-effects models were applied due to the limited number of studies [42, 47], despite moderate-to-high heterogeneity observed across domains (*I²* = 77–97%). In this subgroup, patients with ESP showed significantly higher neuroticism (*MD* = 27.44, 95%*CI* [24.96, 29.92], *Z* = 21.72, *p* < 0.001, *I*^*2*^ = 97%), lower extraversion (*MD*=–6.02, 95%*CI* [–8.57, − 3.47], *Z* = 4.63, *p* < 0.001, *I*^*2*^ = 81%), and conscientiousness (*MD*=–5.53, 95%*CI* [–7.86, − 3.20], *Z* = 4.65, *p* < 0.001, *I*^*2*^ = 77% ). No significant differences emerged for openness or agreeableness. The main results of the meta-analysis are presented in Fig. 2.

Funnel plots were symmetrical for raw‑ and T‑score analyses of all five NEO‑FFI domains but should be interpreted cautiously given ≤ 6 studies per analysis (see Supplementary Figure S2).

Sensitivity analyses restricted to high-quality studies (k = 3) yielded similar point estimates (see Supplementary Figure S3) [37, 40, 46]. Neuroticism reached statistical significance in ESP (*p* < 0.05), while other domains remained non‑significant. Heterogeneity persisted (*I²* >80%), indicating that methodological quality did not account for variability.

### Additional evidence from non-NEO-FFI instruments

Five further studies, using instruments other than the NEO-FFI or lacking the data required for meta-analysis, reported cross-sectional associations.

Using the NEO-PIR, individuals with FEP (*n* = 60) reported higher neuroticism and agreeableness, with lower extraversion and conscientiousness compared to American reference data [2]. A subsequent cross-sectional study replicated these findings and additionally reported higher openness in FEP (*n* = 79) [38]. Similarly, in another observational study (*n* = 96) openness and agreeableness were higher and extraversion lower in FEP, whereas differences in neuroticism and conscientiousness were not significant [48]. Using the TCI in FES (*n* = 33) compared to HCs, an observational study reported higher harm avoidance and lower reward dependence, persistence, self-directedness, and cooperativeness, consistent with elevated emotional reactivity and lower social motivation [50]. Finally, using the ZKPQ, FES (*n* = 50) compared with HCs reported higher neuroticism, aggression–hostility, and lower sociability [45].

### Correlations between personality and clinical features

**Fig. 3:**
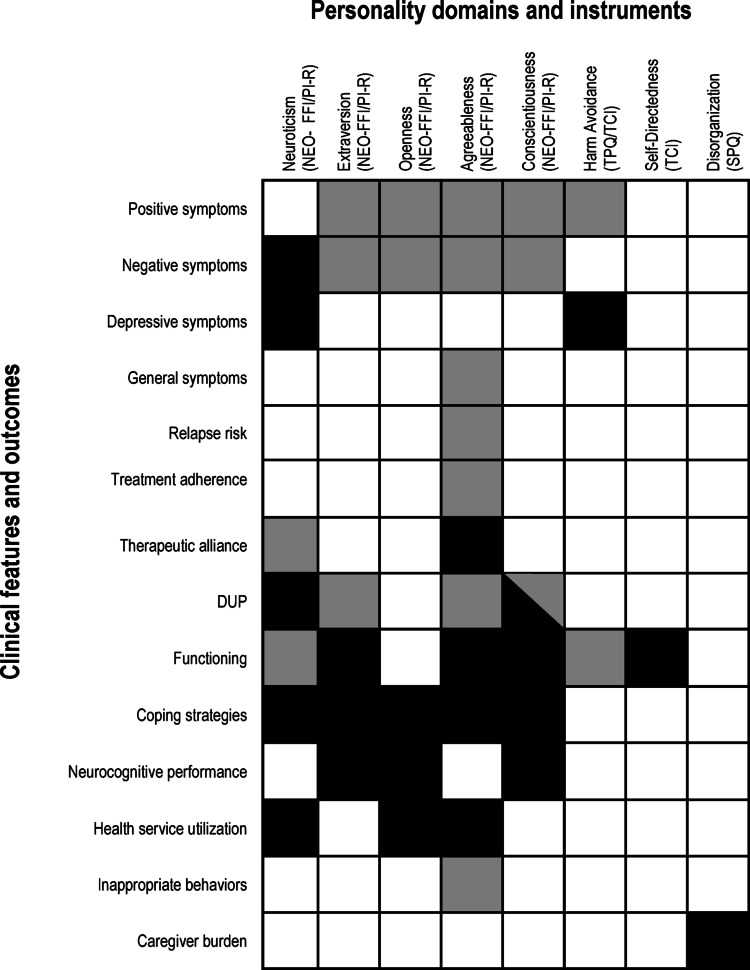
Visual summary of reported associations between personality domains and clinical features in early-stage psychosis in at least one study. Rows correspond to clinical features/outcomes; columns to personality domains and the instruments used. Each cell shows the direction of the reported association. Black square = significant positive association (higher trait level linked to higher outcome score); grey square = significant negative association (higher trait level linked to lower outcome score or vice versa); white square = no significant association reported

Several personality dimensions, assessed using different psychometric instruments, were associated with clinical features in ESP. These associations are described in the following paragraph and visually summarized in Fig. 3 to highlight their consistency across studies. All associations were cross-sectional, except for three studies [2– [39–46] reporting longitudinal findings.

#### Clinical symptoms

Using the NEO-PI-R in FEP, lower extraversion, openness, and conscientiousness were.

associated with greater suspiciousness and unusual thought content [38]. Across NEO-FFI studies, lower agreeableness correlated with increased positive symptom severity, whereas lower extraversion and conscientiousness were associated with negative symptoms in FEP [19]. In ROP, higher agreeableness related to lower overall symptom burden [40], and lower agreeableness, lower extraversion, and higher neuroticism correlated with positive, negative, and depressive clusters, respectively [52]. Neuroticism and conscientiousness positively and negatively correlated, respectively, with negative symptoms in FES [47]. In first-episode of affective psychosis, harm avoidance of the TPQ was positively associated with depressive symptoms and negatively with manic symptoms [44].

In an RCT of FEP, some personality traits (i.e., low agreeableness, low openness, and low conscientiousness) showed significant longitudinal correlations with changes in positive and negative symptoms severity across different conditions, including cognitive-behavioral group therapy (CBT), skills training for symptom management (SM), and a wait-list control group. However, these traits did not significantly predict symptom changes in regression analyses [39]. Two other studies found no association between personality traits and symptom severity [46, 50].

#### Risk of relapse

In a longitudinal study in FEP using the NEO-PI-R, lower agreeableness and its facets (i.e., trust, altruism) predicted higher relapse rates over follow-up [2].

#### Treatment adherence

Higher agreeableness predicted lower adherence to medication in ESP, whereas lower agreeableness and lower neuroticism predicted reduced service engagement [37].

#### Therapeutic alliance

Higher agreeableness and lower neuroticism assessed with the NEO-FFI correlated with stronger patient-rated therapeutic alliance in ROS, while agreeableness also contributed to therapist-rated alliance [42].

#### Duration of untreated psychosis (DUP)

Higher neuroticism and lower extraversion, agreeableness, and conscientiousness were associated with a longer DUP in a NEO-FFI study in FEP [19]. Similar results were observed in another FEP sample using the EPQ, where neuroticism moderated the relationship between social support and DUP: higher neuroticism was linked to longer DUP only in the presence of good social support [49]. A smaller FES study using the BFI-10 replicated that higher conscientiousness was associated with longer DUP [43].

#### Functioning

Functioning was operationalized as the combined score of the Global Assessment of Functioning (GAF) and the Social and Occupational Functioning Assessment Scale (SOFAS). In FEP, extraversion, agreeableness, and conscientiousness assessed with the NEO-FFI correlated positively with overall functioning, whereas neuroticism correlated negatively. However, when adjusting for covariates, no single domain emerged as a unique predictor [19]. In a FES sample, lower harm avoidance and higher self-directedness in the TCI corresponded to better social functioning [50].

#### Coping strategies

In a large FEP sample, conscientiousness, extraversion, openness to experience, and agreeableness longitudinally predicted improvements in active coping, while only neuroticism correlated positively with passive coping [46]. A subsequent study confirmed the positive longitudinal associations with enhanced active coping, while only openness was positively associated with improvements in passive coping [39]. In a broader ROP cohort, higher neuroticism aligned with more passive strategies and less active coping [52].

#### Health service utilization

In a cohort of patients with ROP, openness was directly associated with increased use of ambulatory services [52]. Other traits, such as neuroticism and conscientiousness, showed indirect effects mediated by coping strategies [52].

#### Other clinical outcomes

In a study using the NEO-FFI, agreeableness was inversely related to inappropriate community behaviors (i.e., physical aggression, theft, or illicit drug use) in FEP [48]. In the same sample, neuroticism showed a trend-level association with lower quality of life, although results were not consistent across analytic models [48].

Personality domains assessed with the NEO-FFI were not associated with caregiver burden in ROS. However, disorganization, captured by the SPQ, was independently linked to higher levels of caregiver worry [41].

Regarding neurocognitive abilities in FES, higher extraversion and conscientiousness were correlated with greater attentional and planning abilities, while higher openness to experience was associated with better memory performance [47].

Finally, in FEP patients treated with risperidone, lower levels of agreeableness were associated with higher daily medication dosage [19].

### Assessment of risk of bias and quality of studies

Risk-of-bias ratings are reported in Supplementary Table S5. Both RCTs [39, 46] were judged as low risk overall according to the RoB 2.0 tool. Among the 16 observational studies assessed using the STROBE checklist, scores ranged from 8 to 30 (mean = 18.6 ± 6.7; median = 20). Based on the pre-defined threshold (>17 points), 9 studies were rated as low risk [2, 37, 40, 43, 45, 47, 49, 50, 52], and 7 as high risk. When stratified by analysis type, 1/2 RCTs [46] and 3/7 observational studies included in the meta-analysis were at low risk [37, 40, 47], compared to 6/9 among those contributing to qualitative synthesis [2, 43, 45, 49, 50, 52].

## Discussion

To the best of our knowledge, this is the first systematic review to synthesize the evidence on personality domains and their associations with clinical features in ESP.

Differences in raw scores showed non-significant trends toward higher neuroticism and lower extraversion and conscientiousness in patients, while the meta-analysis based on T-scores revealed that these traits differ significantly compared to HCs. This discrepancy is likely methodological. Harmonized raw scores still originated from heterogeneous sources, inflating between-study variance and reducing statistical power. In contrast, T-scores are age- and sex-standardized against a common reference population, which reduces measurement noise and may better capture differences in personality traits between patients and HCs. Hence, patients with ESP are likely to show increased emotional reactivity and stress sensitivity, as well as reduced social engagement and goal-directed behavior. This pattern replicates findings obtained in patients with chronic psychosis [11, 12] and supports developmental models proposing personality domains as a contributing element in the modulatory pathway to psychosis [1, 53].

Evidence from individuals with CHR-P and unaffected relatives suggests that these traits may represent pre-existing vulnerabilities rather than sequelae of illness [54]. Genomic evidence lends biological plausibility to this interpretation. Indeed, a moderate genetic correlation between neuroticism and schizophrenia was observed (r_g_~0.24–0.26), indicating shared common variant liability [55].

Of note, the results based on raw scores should be interpreted with particular caution, as the very high heterogeneity observed (I²=100%) is likely to reflect substantive differences across studies. In particular, although all samples included ESP patients, they consisted of comorbid groups with different diagnoses, including schizophreniform disorder, schizophrenia, schizoaffective disorder, affective psychoses, substance-induced psychosis, and psychosis not otherwise specified. Further potential moderators include recruitment context (early intervention programs and community clinics [37, 40, 41, 46, 51], vs. inpatient and emergency units [19, 41]), study design (experimental and observational studies, including both cross-sectional and longitudinal designs), and ethnic composition (mostly Caucasian in most cohorts, but predominantly African American in one cohort [51]).

An additional moderator includes the variability in symptom severity, ranging from moderate to marked psychopathology (i.e., brief psychiatric rating scale (BPRS) ~42 in [37–46]), to differences in positive symptoms (Positive and Negative Syndrome Scale [PANSS] – positive >20 [19] vs. ~13 [40]) and negative symptoms (PANSS – negative ~21 [19] vs. ~14 [40]), although comparisons are limited by the use of different assessment tools. Pharmacological treatment and educational attainment likely minimally contributed to heterogeneity, as most patients received second-generation antipsychotics and reported ~12 years of education. The limited variability of control groups, mostly derived from the same normative dataset, may have accentuated patient-side heterogeneity.

From a clinical point of view, albeit based on a limited number of studies, the associations observed with T-scores may suggest that high neuroticism, linked to emotional liability, stress sensitivity, and mood instability, may worsen psychotic symptoms and delay functional recovery [13]. Low extraversion may contribute to early social withdrawal and anhedonia, commonly observed in prodromal and early psychotic phases [56]. Baseline data from a cohort of patients with psychotic disorders indicate that neuroticism accounts for 41% of the variance in personal recovery outcomes, above and beyond symptom severity and treatment exposure, with extraversion contributing an additional 16% [57]. Low conscientiousness may compromise treatment adherence and therapeutic alliance through poor planning, self-discipline, and goal-directed behavior [58].

Although based on preliminary evidence and predominantly cross-sectional evidence, tailored early interventions, such as emotion regulation strategies for high neuroticism [59], social-skills training for low extraversion [60], and motivational approaches for low conscientiousness [61], may represent promising avenues to be tested in prospective interventional studies aimed at preventing maladaptive patterns from consolidation.

In contrast, lower openness and agreeableness appear mainly in chronic stages of schizophrenia [11, 12]. These domains may decline over time because of progressive social withdrawal, stigma, reduced rehabilitative opportunities [62, 63], and long-term antipsychotic effects such as emotional blunting or reduced cognitive flexibility [64]. Notably, most studies included medicated patients, with only three enrolling drug-naïve patients [45, 47, 49], raising the possibility that long-term pharmacological treatment may contribute over time to changes in domains such as openness and agreeableness. Cumulative social cognitive deficits, including impairments in facial emotion recognition or mentalizing capacity, tend to intensify in later illness stages and may further reduce openness and agreeableness [65]. Ideally, comparisons between drug-naïve and medicated cohorts would help clarify medication effects on self-reported traits. However, the limited number of drug-naïve studies, heterogeneity of psychometric instruments, and lack of compatible data precluded stratified analyses.

Among all domains, associations between personality and clinical features were also most consistent for neuroticism, extraversion, and conscientiousness. However, personality did not predict symptom change in multivariate models [39], suggesting a modulatory rather than primary etiological role. This interpretation aligns with stress-vulnerability models, in which dispositional characteristics such as personality interact with neurocognitive functioning, coping strategies, and prior treatment exposure to influence the clinical expression and longitudinal course of psychotic disorders [66]. High neuroticism correlated with negative and depressive symptoms, passive coping, prolonged DUP, and poorer therapeutic alliance. These patterns were also observed in CHR-P and chronic schizophrenia [13, 56] and align with stress-sensitivity models positing that affective lability amplifies internalizing symptomatology. Conversely, low extraversion and conscientiousness were linked to functional decline, avolition, and treatment delay, consistent with their roles in motivational and executive dysfunction [11, 58]. Lower agreeableness was associated with positive symptom severity, relapse risk, and poor therapeutic alliance, although intriguingly some data suggest that high agreeableness may paradoxically undermine medication adherence [37]. This counterintuitive finding may reflect psychosocial or interpersonal mechanisms. Highly agreeable patients may tend to conform to the expectations of significant others. Although this trait may facilitate cooperation with clinicians, it may also increase vulnerability to peer influence or stigma. In contexts where medication use is perceived negatively, high agreeableness might therefore undermine medication adherence. These dynamics highlight the importance of considering the broader social context when interpreting and predicting personality–treatment associations. Openness showed minimal direct associations with symptom severity but was positively associated with greater healthcare utilization, possibly reflecting higher cognitive flexibility and help-seeking behavior. Similar patterns were also reported in non-clinical populations, where high-openness individuals more commonly used conventional and alternative healthcare services [67].

Overall, our findings suggest that personality traits in ESP are clinically relevant characteristics that contribute to variability in symptom expression and clinical outcomes. Routine personality assessment at initial clinical contact may improve clinical decision-making by identifying risk profiles and guiding treatment focus (i.e., stress-targeted interventions for high neuroticism, motivational enhancement for low conscientiousness, and social-skills training for low extraversion). However, the clinical utility of personality-tailored modules within early psychosis programs remains to be established through prospective trials.

### Limitations and future perspectives

Our findings should be interpreted in the context of several limitations. First, given the heterogeneity in the definitions of ESP across studies, we adopted inclusive criteria to reflect real-world variability in how FEP, FES, ROP, ROS, and EP are operationalized in the literature. Second, most included studies were cross-sectional in design, limiting the ability to determine the temporal direction of associations. Third, the paucity of direct patients-HCs comparisons necessitated reliance on single normative samples [8], constraining effect-size precision and precluding meta-regression. These norms were derived from U.S. community samples several decades ago and may not reflect current trait distributions or the cultural background of the patient samples included in the present review. Such cultural and temporal mismatches could bias effect-size estimates, either inflating or attenuating observed differences. Although this approach was the most rigorous option available given the paucity of HC groups in primary studies, findings should be interpreted with caution. Fourth, raw and standardized scores were not consistently reported across studies, requiring separate analyses and reducing the statistical power. Additionally, despite performing sensitivity analyses restricted to high-quality studies, heterogeneity remained substantial (*I²* >80%) and could not be explained by study quality alone. Due to these methodological constraints, the meta-analytic results should be considered exploratory. Finally, as most participants were already on antipsychotic treatment at assessment, a potential medication-related bias cannot be excluded. Dopamine-D₂ antagonism was linked to emotional blunting and neuroleptic dysphoria [68, 69], which may reduce positive affect and motivation, potentially decreasing extraversion and conscientiousness or increasing neuroticism on self-report measures.

Future research should prioritize longitudinal and multimodal designs that follow individuals from the CHR-P through FEP and into disorder consolidation [70]. This would allow for a clearer differentiation between trait vulnerability and state-dependent personality changes. The inclusion of contemporaneous, culturally matched HC groups assessed with the NEO-FFI or comparable dimensional measures is also needed to provide more precise and generalizable effect-size estimates. Incorporating tools such as ecological momentary assessment and passive digital phenotyping may further elucidate the dynamic interplay between enduring personality traits and momentary affective states in predicting relapse and treatment response [71, 72]. Finally, building on interventions with established feasibility in real-world psychiatric settings [73], future trials should investigate whether the integration of modular, personality-tailored components can influence the early course of psychosis. These targeted approaches may help improve symptoms and daily functioning and contribute to more personalized treatment strategies.

## Conclusion

This systematic review and meta-analysis shows that individuals with ESP may have a personality profile characterized by higher neuroticism and lower extraversion and conscientiousness. These traits are associated with symptom severity, functioning, treatment adherence, and service use. Although one multivariate study did not identify personality traits as independent predictors of symptom change [39], current evidence suggests that personality may modulate key aspects of clinical presentation in early psychosis. Early personality domains assessment may facilitate risk stratification and inform targeted psychological strategies, such as emotion regulation strategies for high neuroticism, motivational approaches for low conscientiousness, or social-skills training for low extraversion. Tailoring interventions to individual trait profiles may improve treatment engagement and clinical outcomes, especially during early phases of psychosis.

## Supplementary Information

Below is the link to the electronic supplementary material.

Supplementary Material 1

## Data Availability

All data analyzed in this systematic review and meta-analysis were extracted from publicly available publications. The extracted datasets and all related materials are included in the manuscript and its supplementary files. No new raw data were generated.
