# Supplementary material for: Personality domains in early stages of psychosis: a systematic review and meta-analysis
Source: Eur Arch Psychiatry Clin Neurosci. 2025 Nov 12;276(3):1111–35. doi: 10.1007/s00406-025-02127-4 (PMC13002659; doi:10.1007/s00406-025-02127-4)
Supplement: Supplementary file 1 — Supplementary Material 1 [file 406_2025_2127_MOESM1_ESM.docx]

**Appendix I**

Compared to what was reported in the protocol, we made the following minor deviations:

4) **Search date extension:** While the protocol initially specified that the literature search would be conducted in September 2024, this timeframe was extended until March 2025. This adjustment provided a comprehensive and up-to-date review of the available evidence;

5) **Search strategy:** Minor adjustments were made to the search strategy, including the removal of the term “Ultra-High Risk” from the queries, as studies involving patients at ultra-high risk for psychosis were excluded from the present systematic review and meta-analysis in accordance with the registered protocol.

**Supplementry Table S1:** Search strategy

| Pubmed | ("big five"[Text Word] OR "five factor model"[Text Word] OR extraversion[Text Word] OR introversion[Text Word] OR neuroticism[Text Word] OR openness[Text Word] OR agreeableness[Text Word] OR conscientiousness[Text Word] OR "social inhibition"[Text Word] OR "negative affect"[Text Word] OR "dark triads"[Text Word] OR psychopathy[Text Word] OR narcissism[Text Word] OR Machiavellianism[Text Word] OR schizotypal[Text Word] OR perfectionism[Text Word] OR temperament[Text Word] OR internalisation[Text Word] OR internalization[Text Word] OR personality[Text Word] OR "Personality"[Mesh]) | AND | ("first episode"[Text Word] OR "first episode psychosis"[Text Word] OR FEP[Text Word] OR "first episode schizophrenia"[Text Word] OR "early psychosis"[Text Word] OR "early schizophrenia"[Text Word] OR "recent onset"[Text Word] OR "recent onset psychosis"[Text Word] OR "recent onset schizophrenia"[Text Word]) |
| --- | --- | --- | --- |
| CINAHL | ("big five" OR "five factor model" OR extraversion OR introversion OR neuroticism OR openness OR agreeableness OR conscientiousness OR "social inhibition" OR "negative affect" OR "dark triads" OR psychopathy OR narcissism OR Machiavellianism OR schizotypal OR perfectionism OR temperament OR internalisation OR internalization OR personality OR (MH Personality+)) | AND | ("first episode" OR "first episode psychosis" OR FEP OR "first episode schizophrenia" OR "early psychosis" OR "early schizophrenia" OR "recent onset" OR "recent onset psychosis" OR "recent onset schizophrenia") |
| Web of science | ("big five" OR "five factor model" OR extraversion OR introversion OR neuroticism OR openness OR agreeableness OR conscientiousness OR "social inhibition" OR "negative affect" OR "dark triads" OR psychopathy OR narcissism OR Machiavellianism OR schizotypal OR perfectionism OR temperament OR internalisation OR internalization OR personality) | AND | ("first episode" OR "first episode psychosis" OR FEP OR "first episode schizophrenia" OR "early psychosis" OR "early schizophrenia" OR "recent onset" OR "recent onset psychosis" OR "recent onset schizophrenia") |

**Supplementary Table S2:** Overview of validated psychometric instruments used to assess dimensional personality traits in the included studies. The table summarizes the key characteristics of each tool, including the theoretical model, number of items, and primary personality dimensions assessed. All instruments are based on dimensional approaches to personality and were considered suitable for inclusion in the present systematic review.

| **Tool** | **Framework** | **Format & Properties** | **Dimensions** | **Interpretation** |
| --- | --- | --- | --- | --- |
| **Tridimensional Personality Questionnaire (TPQ)** | Cloninger's Three-Factor Model of Temperament | 100 items  dichotomous responses (Yes/No) | Novelty Seeking (NS)  Harm Avoidance (HA)    Reward Dependence (RD) | The TPQ is based on Cloninger’s psychobiological model, which identifies three temperament traits as biologically independent, early-emerging dimensions that influence automatic behavioral responses. These traits are associated with behavioral activation, sensitivity to aversive stimuli, and responsiveness to social rewards, respectively. |
| **NEO Personality Inventory-Revised (NEO-PI-R)** | Costa and McCrae's Five-Factor Model | 240 items, 5-point Likert scale | Neuroticism (N)  Extraversion (E)  Openness to Experience (O)  Agreeableness (A)  Conscientiousness (C) | The NEO-PI-R assesses the Five-Factor Model of personality:  N=emotional instability and vulnerability to psychological distress  E=degree of sociability, assertiveness, and positive affect  O=cognitive flexibility, curiosity, and receptiveness to novel ideas and experiences  A=tendencies toward altruism, empathy, and interpersonal cooperation  C=traits related to organization, goal-directed behavior, and self-discipline  Each domain is measured through six lower-order facets, allowing for detailed profiling. The instrument provides a robust empirical framework for evaluating individual differences across broad personality dimensions. |
| **NEO Five-Factor Inventory (NEO-FFI)** | Costa and McCrae's Five-Factor Model | 60 items, 5-point Likert scale | Neuroticism (N)  Extraversion (E)  Openness to Experience (O)  Agreeableness (A)  Conscientiousness (C) | The NEO-FFI is a shortened version of the NEO-PI-R and measures the same five personality domains without assessing facet-level information. It is suitable for contexts requiring efficient, time-constrained personality assessments, maintaining adequate reliability and construct validity for large-scale applications. |
| **Eysenck Personality Questionnaire (EPQ)** | Eysenck's Psychobiological Model  (Three-Factor Theory) | 90 items in the adult versión  48 in the short version  dichotomous responses (Yes/No) | Psychoticism  Extraversion  Neuroticism | The EPQ reflects Eysenck’s biologically oriented theory of personality, which includes the dimensions of Psychoticism, Extraversion, and Neuroticism. These traits are believed to correspond to physiological arousal systems and account for individual differences in impulsivity, sociability, and emotional reactivity. |
| **Temperament and Character Inventory (TCI)** | Cloninger's Psychobiological Model | 240 items  dichotomous responses (Yes/No) | Novelty Seeking  Harm Avoidance  Reward Dependence  Persistence  Self-Directedness  Cooperativenes  Self-Transcendence | The TCI extends the TPQ by including character dimensions in addition to temperament. Temperament is described as biologically influenced and expressed through habitual emotional responses, whereas character refers to higher-order self-regulatory traits shaped by individual goals, values, and social learning.  This structure allows for a more comprehensive assessment of personality development across both innate and adaptive domains. |
| **Zuckerman–Kuhlman Personality Questionnaire (ZKPQ)** | Zuckerman's Alternative Five-Factor Model | 99 items  dichotomous responses (Yes/No) | Impulsivity-Sensation Seeking  Sociability  Neuroticism-Anxiety  Aggression-Hostility  Activity | The ZKPQ is designed to evaluate five dimensions derived from biosocial theory. The model emphasizes biological and temperamental influences on personality traits and differentiates between anxiety and aggression-related dimensions, offering an alternative to the traditional Five-Factor Model. |
| **Big Five Inventory-10 Item Scale (BFI-10)** | Big Five Factor Model | 10 items  5-point Likert scale | Neuroticism (N)  Extraversion (E)  Openness to Experience (O)  Agreeableness (A)  Conscientiousness (C) | The BFI-10 offers a brief yet psychometrically acceptable measure of the Big Five personality traits. It is intended for use in research contexts where rapid assessment is essential, such as large-scale surveys or field studies, and retains theoretical alignment with the Five-Factor framework despite its brevity. |
| **Schizotypal Personality Questionnaire (SPQ)** | DSM-III-R criteria for Schizotypal Personality Disorder | 74 items  dichotomous responses (Yes/No) | Positive schizotypy (ideas of reference, magical thinking, unusual perceptual experiences)  Negative schizotypy  (social anxiety, anhedonia, constricted affect)  Disorganization (eccentric behavior, odd speech). | The SPQ assesses schizotypal personality traits dimensionally, based on the DSM-III-R criteria for Schizotypal Personality Disorder. Despite its diagnostic origins, the SPQ is widely used in non-clinical and clinical populations as a dimensional measure of schizotypy. Psychometric evaluations indicate a robust three-factor structure with good convergent and discriminant validity |

**Supplementary Table S3:** Studies subject to full-text review but excluded from systematic review and meta-analysis

| **Authors** | **Title** | **Published Year** | **Reason** |
| --- | --- | --- | --- |
| **Archer, M. et al.** | Mentalizing in first-episode psychosis: Correlates with symptomatology and traits of borderline personality disorder. | 2022 | Ineligible outcome |
| **Azzali, A. et al.** | Examining subjective experience of aberrant salience in young individuals at ultra-high risk (UHR) of psychosis: A 1-year longitudinal study | 2022 | Ineligible outcome and population |
| **Barch, D.et al.** | Intrinsic motivation in schizophrenia: relationships to cognitive function, depression, anxiety, and personality | 2008 | Ineligible outcome and population |
| **Bastiaens, T. et al.** | Self-Reported Cognitive Biases Are Equally Present in Patients Diagnosed With Psychotic Versus Nonpsychotic Disorders | 2018 | Ineligible outcome and population |
| **Bastiaens, T. et al.** | The Relationship Between the Personality Inventory for the DSM-5 (PID-5) and the Psychotic Disorder in a Clinical Sample | 2019 | Ineligible outcome and population |
| **Betensky, J. et al.** | Patterns of stress in schizophrenia | 2008 | Ineligible outcome and population |
| **Boyette, L. et al.** | Quality of life in patients with psychotic disorders: impact of symptoms, personality, and attachment | 2014 | Ineligible population |
| **Boyette, L. et al.** | Personality Compensates for Impaired Quality of Life and Social Functioning in Patients With Psychotic Disorders Who Experienced Traumatic Events | 2014 | Ineligible outcome and population |
| **Boyette, L. et al.** | Neuroticism and Extraversion are modifiable by treatment in individuals at-risk for psychosis or with first-episode psychotic disorder | 2024 | Ineligible population |
| **Browne, J. et al.** | Character strengths of individuals with first episode psychosis in Individual Resiliency Training | 2018 | Ineligible outcome |
| **Campos, M. et al.** | Premorbid personality and insight in first-episode psychosis | 2011 | Ineligible outcome |
| **Canal-Rivero, M. et al.** | One-year follow-up study of first suicide attempts in first episode psychosis: Personality traits and temporal pattern | 2016 | Ineligible outcome |
| **Canal-Rivero, M. et al.** | Suicidal Behavior and Personality Traits Contribute to Disability in First-episode Psychosis: A 1-Year Follow-up Study | 2019 | Ineligible outcome |
| **Canal-Rivero, M. et al.** | The role of premorbid personality and social cognition in suicidal behaviour in first-episode psychosis: A one-year follow-up study | 2017 | Ineligible outcome |
| **Canal-Rivero, M. et al.** | Personality traits, theory of mind and their relationship with multiple suicide attempts in a sample of first episode psychosis patients: One-year follow-up study | 2019 | Ineligible outcome |
| **Carter, J. et al.** | Identifying non-affective psychosis in first admission patients: MMPI-2, structured diagnostic interview, and consensus lifetime best estimate | 2019 | Ineligible outcome |
| **Castelein, S. et al.** | Suicide in Recent Onset Psychosis Revisited: Significant Reduction of Suicide Rate over the Last Two Decades - A Replication Study of a Dutch Incidence Cohort | 2015 | Ineligible outcome |
| **Chen, C. et al.** | The relationship between resting electroencephalogram oscillatory abnormalities and schizotypal personality traits in the first-degree relatives of schizophrenia patients | 2019 | Ineligible outcome and population |
| **Cuesta, M. et al.** | Premorbid personality and psychopathological dimensions in first-episode psychosis | 2002 | Ineligible outcome |
| **Cuesta, M. et al.** | Can insight be predicted in first-episode psychosis patients? A longitudinal and hierarchical analysis of predictors in a drug-naïve sample | 2011 | Ineligible outcome |
| **Cuesta, M. et al.** | Premorbid negative symptoms in first-episode psychosis | 2007 | Ineligible outcome |
| **De Page, L. and Titeca P.** | Patients are changing - The ripple effects of changing the law and creating new forensic beds in Belgium | 2021 | Ineligible outcome and population |
| **De Salve, F. et al.** | Personality traits and transition to psychosis one year after the first assessment | 2023 | Ineligible outcome |
| **Demro, C. et al.** | Advanced Brain-Age in Psychotic Psychopathology: Evidence for Transdiagnostic Neurodevelopmental Origins | 2022 | Ineligible outcome and population |
| **Drvaric, L. et al.** | Maladaptive personality traits in patients identified at lower-risk and higher-risk for psychosis | 2018 | Ineligible population |
| **W. S. Edell** | Relationship of borderline syndrome disorders to early schizophrenia on the MMPI | 1987 | Ineligible outcome |
| **Esposito, C. et al.** | First episode psychosis during the Covid-19 pandemic in Milan, Italy: Diagnostic outcomes at 1-year follow-up | 2023 | Ineligible outcome |
| **Feola, B. et al.** | Evidence for inhibited temperament as a transdiagnostic factor across mood and psychotic disorders | 2020 | Ineligible outcome and population |
| **Flückiger, R. et al.** | Psychosis-predictive value of self-reported schizotypy in a clinical high-risk sample | 2016 | Ineligible outcome and population |
| **Fresán, A. et al.** | Personality features in ultra-high risk for psychosis: a comparative study with schizophrenia and control subjects using the Temperament and Character Inventory-Revised (TCI-R) | 2015 | Ineligible population |
| **Gajwani, R. et al.** | Recruiting and exploring vulnerabilities among young people at risk, or in the early stages of serious mental illness (borderline personality disorder and first episode psychosis) | 2022 | Ineligible outcome |
| **Gerritsen, C. et al.** | Personality traits in psychosis and psychosis risk linked to TSPO expression: a neuroimmune marker | 2020 | Ineligible outcome |
| **Gheorge, M. et al.** | Premorbid cognitive and behavioral functioning in military recruits experiencing the first episode of psychosis | 2004 | Ineligible outcome |
| **Gruber, M. et al.** | Personality functioning and self-disorders in individuals at ultra-high risk for psychosis, with first-episode psychosis and with borderline personality disorder | 2023 | Ineligible outcome |
| **Guerrero-Jiménez, M. et al.** | A cross-sectional survey of psychotic symptoms in the community: The GRANADΣP psychosis study | 2018 | Ineligible outcome and population |
| **M. Hambrecht** | Factors influencing the validity of relatives' reports about symptoms of first-episode schizophrenia | 1997 | Ineligible outcome and population |
| **Hasson-Ohayon,I. et al.** | Insight, personality, and symptoms among individuals with psychosis: Cross-sectional and longitudinal relationships | 2020 | Ineligible outcome and population |
| **Hayes, J. et al.** | Association of Late Adolescent Personality With Risk for Subsequent Serious Mental Illness Among Men in a Swedish Nationwide Cohort Study | 2017 | Ineligible outcome and population |
| **Hegde, A. et al.** | Caregiver distress in schizophrenia and mood disorders: the role of illness-related stressors and caregiver-related factors | 2019 | Ineligible outcome and population |
| **Heikkilä, J. et al.** | Psychodynamic personality profile in first-episode severe mental disorders | 2004 | Ineligible outcome |
| **Hogg, B. and et al.** | Diagnosing Personality-Disorders in Recent Onset Schizophrenia | 1990 | Ineligible outcome |
| **Horan, W. et al.** | Stability and clinical correlates of personality characteristics in recent-onset schizophrenia | 2005 | Ineligible outcome |
| **Hui, C. et al.** | Delusional disorder and schizophrenia: a comparison of the neurocognitive and clinical characteristics in first-episode patients | 2015 | Ineligible outcome |
| **Hui, C. et al.** | Examining gender difference in adult-onset psychosis in Hong Kong | 2016 | Ineligible outcome |
| **Hui, C. et al.** | Predictors of help-seeking duration in adult-onset psychosis in Hong Kong | 2013 | Ineligible outcome |
| **Kotlicka-Antczak, M. et al.** | A developmentally-stable pattern of premorbid schizoid-schizotypal features predicts psychotic transition from the clinical high-risk for psychosis state | 2019 | Ineligible outcome and population |
| **Ku, B. et al.** | Residential instability during adolescence predicts earlier age at onset of psychosis: The moderating role of extraversion | 2023 | Ineligible population |
| **Liemburg, E. et al.** | The Psychosis Recent Onset GRoningen Survey (PROGR-S): defining dimensions and improving outcomes in early psychosis | 2014 | Ineligible outcome |
| **Liu, J. et al.** | Anticipating Unipolar Depression and Bipolar Depression in young adult with first episode of depression using childhood trauma and personality | 2022 | Ineligible outcome and population |
| **Longenecker, J. et al.** | Personality traits across the psychosis spectrum: A Hierarchical Taxonomy of Psychopathology conceptualization of clinical symptomatology | 2020 | Ineligible outcome and population |
| **Lovretic, V. et al.** | Association of Childhood Trauma and Personality Dimensions with the Duration of Untreated Psychosis | 2022 | Ineligible outcome |
| **Mamah, D. et al.** | Personality Traits as Markers of Psychosis Risk in Kenya: Assessment of Temperament and Character | 2020 | Ineligible population |
| **Marshall C et al.** | Treating young individuals at clinical high risk for psychosis | 2012 | Ineligible population |
| **Mason, O. et al.** | Risk factors for transition to first episode psychosis among individuals with 'at-risk mental states' | 2004 | Ineligible outcome and population |
| **Meliante, M. et al.** | The Relationship between PID-5 Personality Traits and Mental States. A Study on a Group of Young Adults at Risk of Psychotic Onset | 2021 | Ineligible population |
| **Miralles, C. et al.** | Maladaptive personality traits in patients with recent-onset psychosis: A case-control study using the Personality Inventory for the DSM-5 (PID-5) | 2023 | Ineligible population |
| **Morrison, A. et al.** | Randomised controlled trial of early detection and cognitive therapy for preventing transition to psychosis in high-risk individuals - Study design and interim analysis of transition rate and psychological risk factors | 2002 | Ineligible outcome and population |
| **Nishiyama, S. et al.** | Development and validation of a scale of self-alienation-related attributes for the early diagnosis of schizophrenia | 2022 | Ineligible outcome and population |
| **Pelizza, L. et al.** | Anhedonia in the Psychosis Risk Syndrome: State and Trait Characteristics | 2021 | Ineligible outcome and population |
| **Pionke-Ubych, R. et al.** | Integrating trauma, self-disturbances, cognitive biases, and personality into a model for the risk of psychosis: a longitudinal study in a non-clinical sample | 2022 | Ineligible outcome and population |
| **Ramsay, C. et al.** | Overview and initial validation of two detailed, multidimensional, retrospective measures of substance use: the Lifetime Substance Use Recall (LSUR) and Longitudinal Substance Use Recall for 12 Weeks (LSUR-12) Instruments | 2011 | Ineligible outcome and population |
| **Rossi, C. et al.** | At-risk mental states and personality traits: A cluster analysis approach on a group of help-seeking young adults | 2023 | Ineligible population |
| **Rossini. L. et al.** | Salivary cortisol response to psychosocial stress in patients with first-episode psychosis | 2021 | Ineligible outcome |
| **Sevilla-Llewellyn-Jones, J. et al.** | Personality traits in recent-onset-of-psychosis patients compared to a control sample by gender | 2018 | Ineligible outcome |
| **Sevilla-Llewellyn-Jones, J.** | Personality Traits and Psychotic Symptoms in Recent Onset of Psychosis Patients | 2018 | Ineligible outcome |
| **Sevilla-Llewellyn-Jones, J. et al.** | Subjective quality of life in recent onset of psychosis patients and its association with sociodemographic variables, psychotic symptoms and clinical personality traits | 2019 | Ineligible outcome |
| **Sevilla-Llewellyn-Jones, J. et al.** | Personality traits and psychotic symptoms in recent onset of psychosis patients | 2017 | Ineligible outcome |
| **Sotiropou M et al.** | BDNF serum concentrations in first psychotic episode drug-naïve schizophrenic patients: Associations with personality and BDNF Val66Met polymorphism | 2013 | Ineligible outcome |
| **Strakowski, S. et al.** | The Tridimensional Personality Questionnaire as a Predictor of 6-Month Outcome in 1st Episode Mania | 1993 | Ineligible population |
| **Subotnik, K. et al.** | Trait versus state aspects of the MMPI during the early course of schizophrenia | 1999 | Ineligible outcome |
| **Subotnik, K. et al.** | MMPI discriminators of deficit vs. non-deficit recent-onset schizophrenia patients | 2000 | Ineligible outcome |

**Supplementry Table S4:** Studies using the NEO-FFI excluded from the meta-anlysis.

| **Authors** | **Title** | **Published Year** | **Reason** |
| --- | --- | --- | --- |
| **Couture S et al.** | Personality characteristics and attachment in first episode psychosis: impact on social functioning | 2007 | Excluded due to absence of raw mean and standard deviation values for NEO-FFI domains. |
| **Beauchamp M et al.** | Do personality traits matter when choosing a group therapy for early psychosis? | 2013 | Excluded due to sample overlap with Beauchamp M et al. (2011), already included in the meta-analysis. |
| **Scholte-Stalenhoef A**  **et al.** | Personality traits in psychotic illness and their clinical correlates: a systematic review | 2016 | Excluded due to sample overlap with Djordjevic et al. (2022), already included in the meta-analysis. |

**Supplementary Figure S1:** Sensitivity analyses of studies reporting raw NEO-FFI scores. Forest plots showing pooled mean differences (MDs, 95% CI) between early stage psychosis (ESP) and healthy controls (HC) across the five NEO-FFI personality domains, analyzed separately for studies using the 0–4 Likert format (2 studies) and the 1–5 Likert format (4 studies).

**Response format (Likert 0–4)**

**
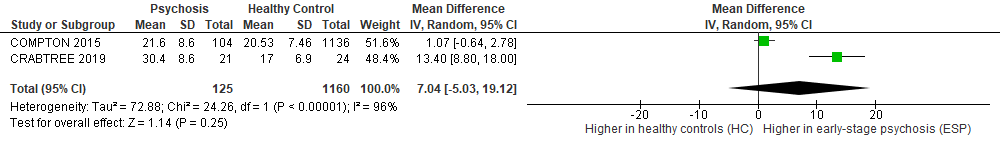
**

Neuroticism (N) – Raw scores

**
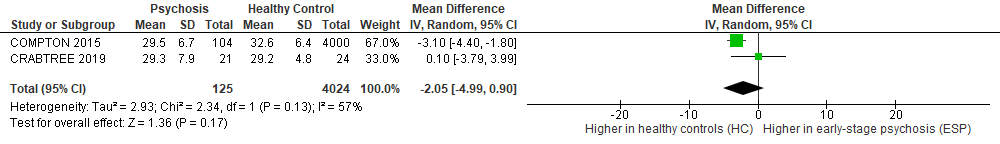
**

Extraversion (E) – Raw scores

**
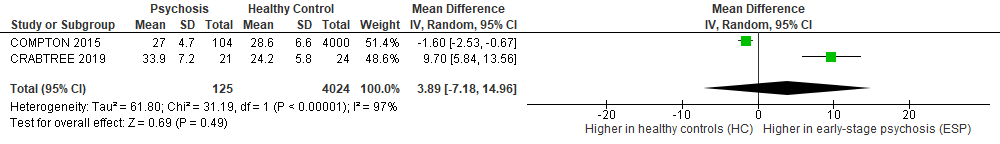
**

Openness to experience (O) – Raw scores

**
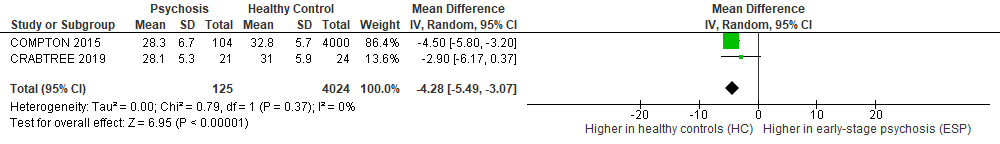
**

Agreeableness (A) – Raw scores

**
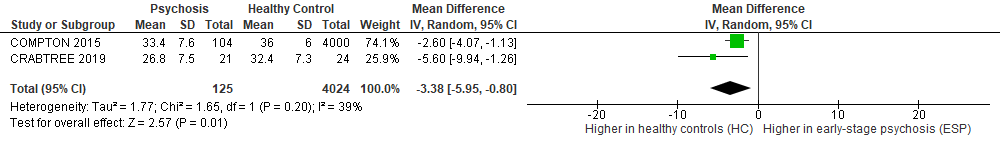
**

Conscientiousness (C) – Raw scores

**Response format (Likert 1–5)**

**
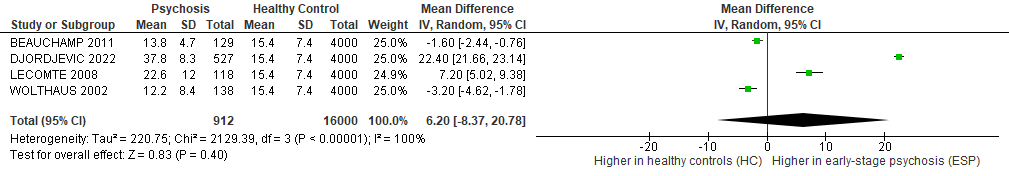
**

Neuroticism (N) – Raw scores

**
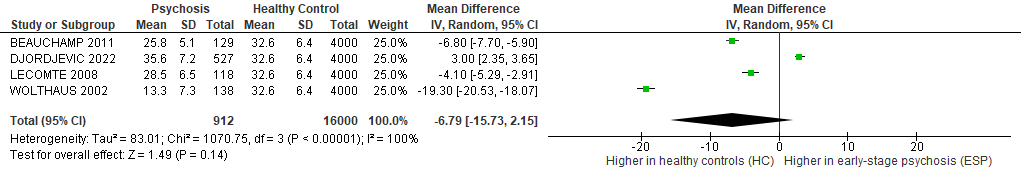
**

Extraversion (E) – Raw scores

**
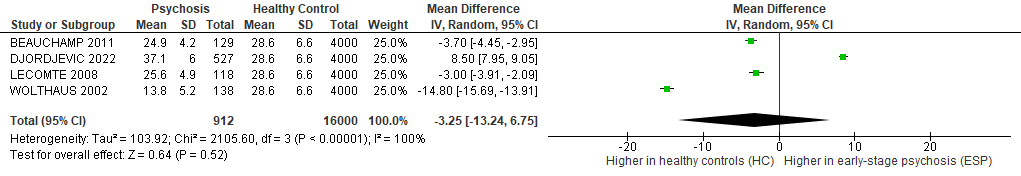
**

Openness to experience (O) – Raw scores

**
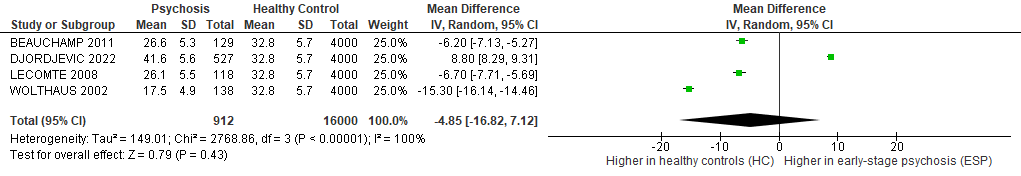
**

Agreeableness (A) – Raw scores

**
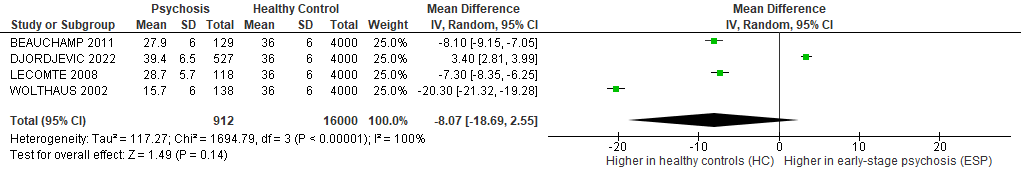
**

Conscientiousness (C) – Raw scores

**Supplementary Figure S2:** Funnel plots evaluating small‑study effects for each NEO‑FFI domain based on raw mean scores and standardized T‑scores for Neuroticism (N), Extraversion (E), Openness to experience (O), Agreeableness (A), and Conscientiousness (C). Each dot represents an individual study; the vertical solid line marks the pooled mean difference, and the diagonal dashed lines indicate pseudo 95 % confidence limits around that estimate. Visual inspection revealed no marked asymmetry in any panel, suggesting an absence of major small‑study effects, although interpretation is limited by the small number of studies (≤ 6 per funnel).

| 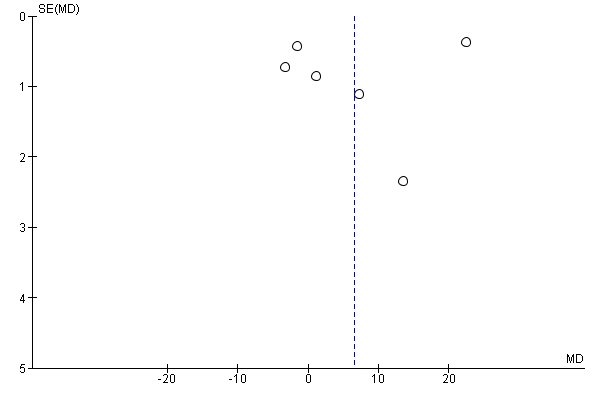  Neuroticism (N) – Raw scores | 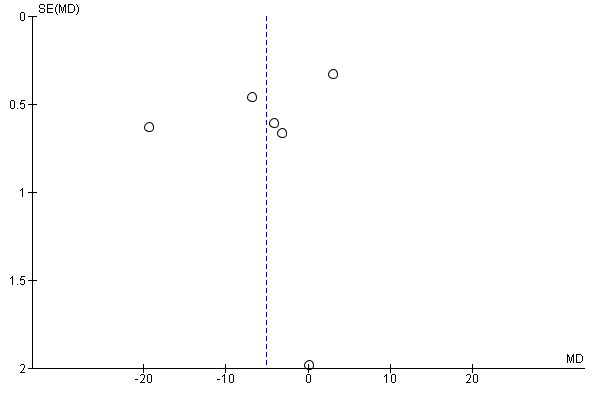  Extraversion (E) – Raw scores |
| --- | --- |
| 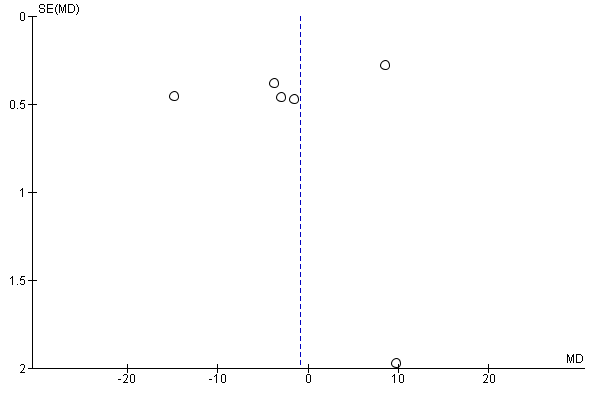Openness to experience (O) – Raw scores | 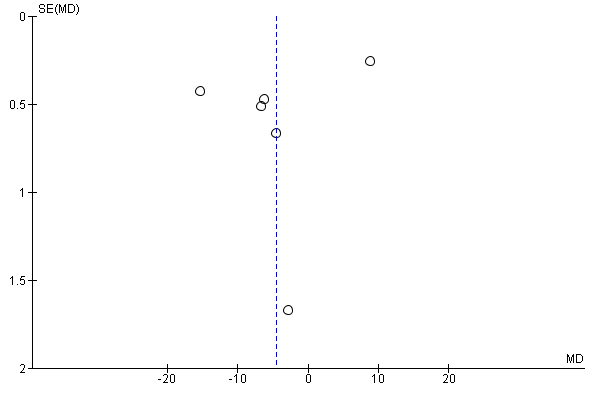  Agreeableness (A) – Raw scores |
| 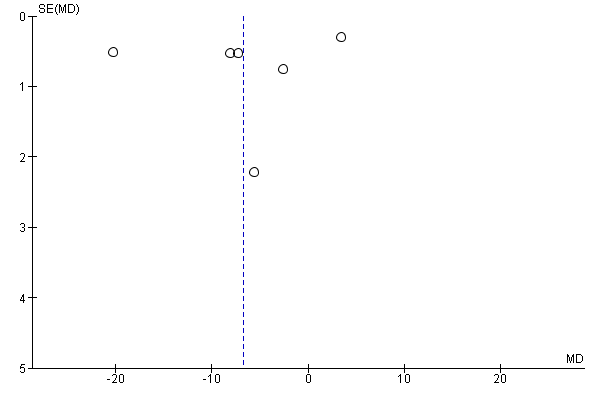 Conscientiousness (C) – Raw scores |  |
| 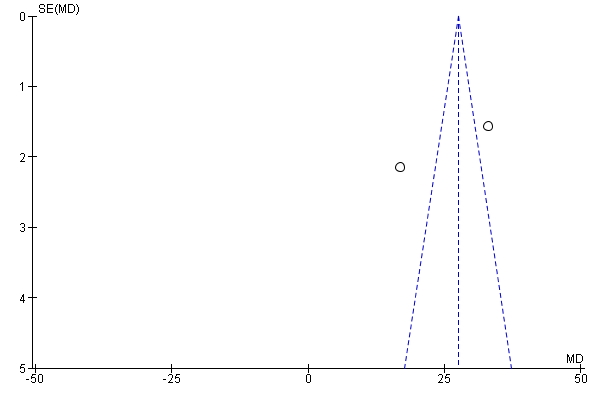 Neuroticism (N) – T‑scores | 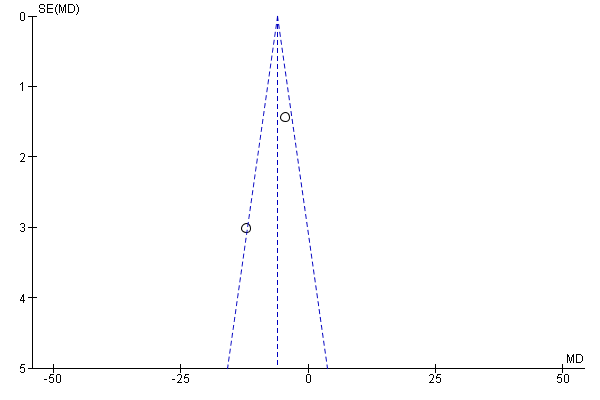 Extraversion (E) – T‑scores |
| 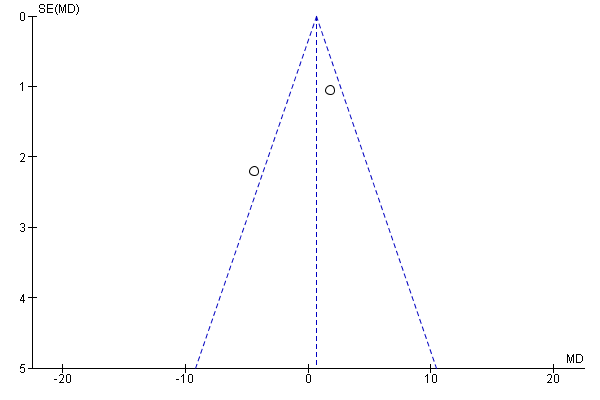 Openness to experience (O) – T‑scores | 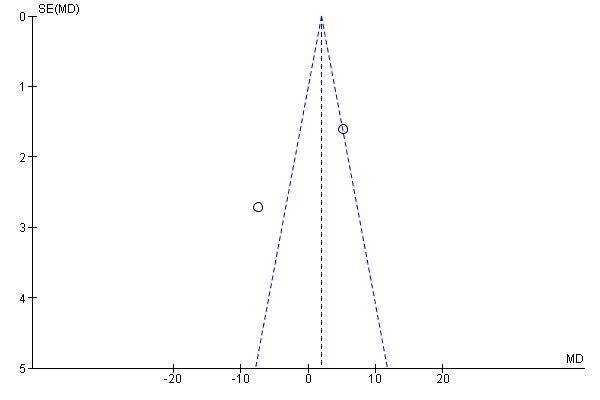 Agreeableness (A) – T‑scores |
| 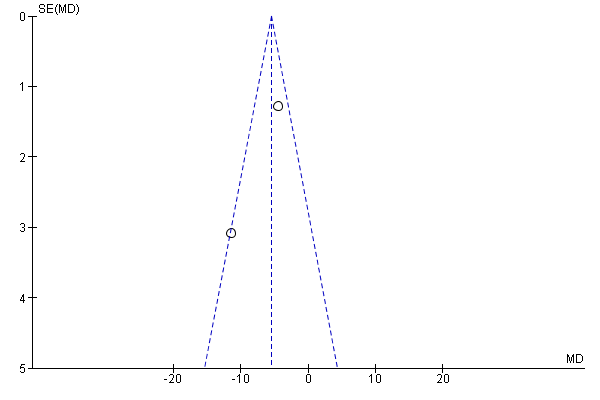 Conscientiousness (C) – T‑scores |  |

**Supplementary Figure S3:** Random‑effects model meta‑analyses for Neuroticism (A), Extraversion (B), Openness (C), Agreeableness (D), and Conscientiousness (E), limited to studies rated as high methodological quality. Symbols indicate study‑specific mean differences (squares, size proportional to study weight) with 95 % confidence intervals. Diamonds denote pooled effects. The pooled estimate for Neuroticism was statistically higher in early‑stage psychosis than in healthy controls, whereas the other domains remained non‑significant. Between‑study heterogeneity persisted at a high level (I² > 80 % in all panels). A sensitivity analysis for standardized T‑scores could not be performed because only two studies (one high‑ and one low‑quality) were available.

| 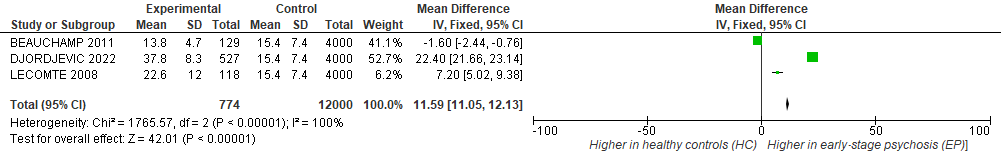  Neuroticism (N) – Raw scores |
| --- |
| 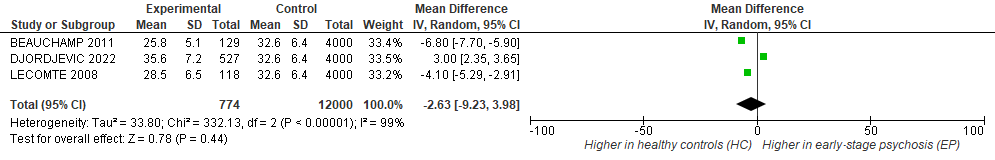  Extraversion (E) – Raw scores |
| 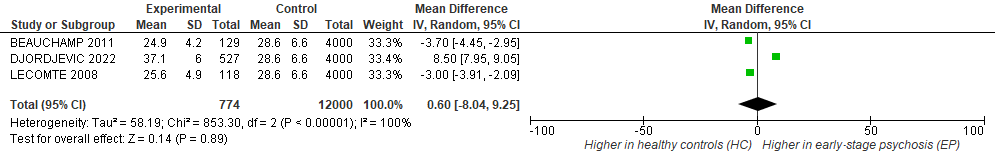  Openness to experience (O) – Raw scores |
| 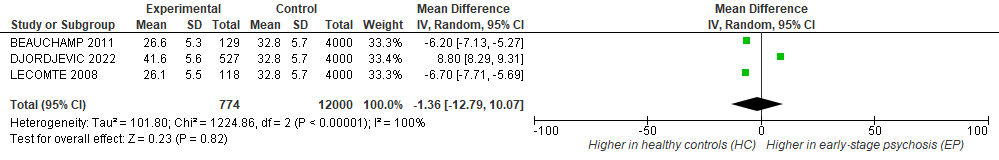  Agreeableness (A) – Raw scores |
| 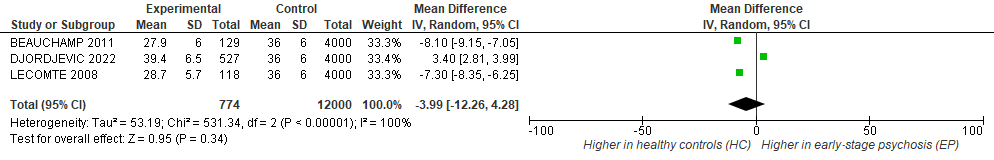  Conscientiousness (C) – Raw scores |

**Supplementary Table S5:** Risk of bias and methodological quality assessment for included studies. Randomized controlled trials were assessed using the Cochrane Risk of Bias Tool 2.0. Observational studies were evaluated using the STROBE Statement Checklist (max score = 34). Studies scoring ≤17 were classified as lower quality (i.e., high risk of bias), while those scoring >17 were considered higher quality.

| **TYPE OF STUDY** | **First author, year** | **RANDOM SEQUENCE GENERATION** | **ALLOCATION CONCEALMENT** | **BLINDING OF PARTICIPANTS** | **BLINDING OF PERSONNEL** | **BLINDING OF OUTCOME ASSESSMENT** | **INCOMPLETE OUTCOME DATA** | **SELECTIVE REPORTING** | **ROB COCHRANE TOOL** |  |
| --- | --- | --- | --- | --- | --- | --- | --- | --- | --- | --- |
| **RCT included in the SR** | **Beauchamp M et al., 2013** | 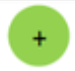 | 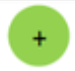 | 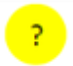 | 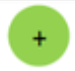 | 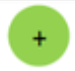 | 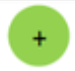 | 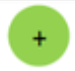 | LOW ROB |  |
| **RCT included in the MA** | **Beauchamp M et al., 2011** | 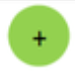 | 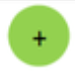 | 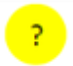 | 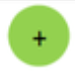 | 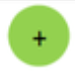 | 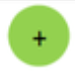 | 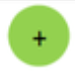 | LOW ROB |  |
|  |  | **TITLE** | **INTRO** | **METHODS** | **RESULTS** | **DISCUSSION** | **OTHER** |  | **TOTALE STROBE STATEMENT** | |
| **OS included in the MA** | **Compton M**  **et al., 2015** | 1 | 1 | 3 | 4 | 3 | 1 | TOTAL | 13 | 38,2352941  HIGH ROB |
|  | **Crabtree et al., 2019** | 0 | 1 | 2 | 1 | 3 | 1 | TOTAL | 8 | 23,5294118  HIGH ROB |
|  | **Djordjevic M**  **et al., 2022** | 1 | 2 | 10 | 6 | 4 | 1 | TOTAL | 24 | 70,5882353  LOW ROB |
|  | **Gurrera R**  **et al., 2014** | 1 | 2 | 8 | 6 | 4 | 1 | TOTAL | 22 | 64,7058824  LOW ROB |
|  | **Johansen R**  **et al., 2013** | 1 | 1 | 5 | 5 | 3 | 1 | TOTAL | 16 | 47,0588235  HIGH ROB |
|  | **Lecomte T**  **et al., 2008** | 2 | 2 | 6 | 5 | 3 | 1 | TOTAL | 19 | 55,8823529  LOW ROB |
|  | **Wolthaus J**  **et al., 2002** | 0 | 1 | 3 | 4 | 2 | 0 | TOTAL | 10 | 29,4117647  HIGH ROB |
| **OS included in the SR** | **Beauchamp M et al., 2006** | 1 | 2 | 4 | 2 | 2 | 1 | TOTAL | 12 | 35,2941176  HIGH ROB |
|  | **Bozidis P**  **et al., 2014** | 2 | 2 | 8 | 5 | 3 | 1 | TOTAL | 21 | 61,7647059  LOW ROB |
|  | **Couture S**  **et al., 2007** | 1 | 1 | 7 | 4 | 3 | 1 | TOTAL | 17 | 50  HIGH ROB |
|  | **Gleeson J**  **et al., 2005** | 2 | 2 | 9 | 8 | 3 | 1 | TOTAL | 25 | 73,5294118  LOW ROB |
|  | **Jo A**  **et al., 2021** | 0 | 1 | 8 | 10 | 3 | 1 | TOTAL | 23 | 67,6470588  LOW ROB |
|  | **Ruiz-Veguilla M**  **et al., 2012** | 1 | 2 | 7 | 9 | 2 | 1 | TOTAL | 22 | 64,7058824  LOW ROB |
|  | **Scholte-Stalenhoef A**  **et al., 2016** | 2 | 2 | 12 | 10 | 3 | 1 | TOTAL | 30 | 88,2352941  LOW ROB |
|  | **Song YY et al., 2013** | 2 | 2 | 11 | 8 | 3 | 1 | TOTAL | 27 | 79,4117647  LOW ROB |
|  | **Strakowski S et al., 1992** | 0 | 1 | 3 | 4 | 1 | 0 | TOTAL | 9 | 26,4705882  HIGH ROB |
|  |  |  |  |  |  |  |  |  |  |  |
|  | **MAXIMUM SCORE** | 2 | 2 | 14 | 11 | 4 | 1 | TOTAL | 34 |  |

**Abbreviations:** MA=meta-analysis; OS= observational study; RCT= randomized clinical trial; ROB= risk of bias; SR=systematic review.
